# Dark and Bright Excitons in Halide Perovskite Nanoplatelets

**DOI:** 10.1002/advs.202103013

**Published:** 2021-12-23

**Authors:** Moritz Gramlich, Michael W. Swift, Carola Lampe, John L. Lyons, Markus Döblinger, Alexander L. Efros, Peter C. Sercel, Alexander S. Urban

**Affiliations:** ^1^ Nanospectroscopy Group Nano‐Institute Munich Department of Physics Ludwig‐Maximilians‐Universität München (LMU) Munich 80539 Germany; ^2^ Center for Computational Materials Science U.S. Naval Research Laboratory Washington D.C. 20375 USA; ^3^ Department of Chemistry Ludwig‐Maximilians‐Universität München (LMU) & Center for NanoScience (CeNS) Munich 81377 Germany; ^4^ Center for Hybrid Organic Inorganic Semiconductors for Energy Golden CO 80401 USA

**Keywords:** effective mass model, exciton fine structure, halide perovskites, nanoplatelets, optoelectronics, photoluminescence spectroscopy, quantum confinement

## Abstract

Semiconductor nanoplatelets (NPLs), with their large exciton binding energy, narrow photoluminescence (PL), and absence of dielectric screening for photons emitted normal to the NPL surface, could be expected to become the fastest luminophores amongst all colloidal nanostructures. However, super‐fast emission is suppressed by a dark (optically passive) exciton ground state, substantially split from a higher‐lying bright (optically active) state. Here, the exciton fine structure in 2–8 monolayer (ML) thick Cs_*n* − 1_Pb_*n*_Br_3*n* + 1_ NPLs is revealed by merging temperature‐resolved PL spectra and time‐resolved PL decay with an effective mass model taking quantum confinement and dielectric confinement anisotropy into account. This approach exposes a thickness‐dependent bright–dark exciton splitting reaching 32.3 meV for the 2 ML NPLs. The model also reveals a 5–16 meV splitting of the bright exciton states with transition dipoles polarized parallel and perpendicular to the NPL surfaces, the order of which is reversed for the thinnest NPLs, as confirmed by TR‐PL measurements. Accordingly, the individual bright states must be taken into account, while the dark exciton state strongly affects the optical properties of the thinnest NPLs even at room temperature. Significantly, the derived model can be generalized for any isotropically or anisotropically confined nanostructure.

## Graphical abstract

2D semiconductor nanoplatelets can enable faster and more efficient light‐emitting devices. For this, a detailed understanding of their energetic structure is paramount. Here, the thickness‐dependent exciton fine structure of nanoplatelets is deduced by merging temperature and time‐resolved photoluminescence spectroscopy with a novel effective mass model, considering anisotropic quantum and dielectric confinement. Relevantly, the model can be generalized for any nanostructure.

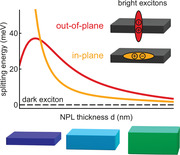

## 1 Introduction

The growing interest in colloidal nanoplatelets (NPLs), which are atomically flat, quasi‐2D sheets of semiconductors, is justified by their potential to become the best luminophores amongst all colloidal nanostructures. The lateral size of the typically rectangular‐shaped NPLs is much larger than their thickness, which can be varied in a precisely controlled manner from two to almost a dozen monolayers (MLs).^[^ ^1^ ^]^ Resulting NPL samples are remarkably uniform and nearly monodisperse in the predetermined thickness. The growth of colloidal quantum wells, such as the NPLs or nanoribbons was first reported for II–VI compounds (e.g., CdSe, CdTe, CdS)^[^ ^2^, ^3^, ^4^ ^]^ and subsequently for lead halide perovskites.^[^ ^5^, ^6^, ^7^, ^8^, ^9^, ^10^ ^]^ Optical investigations show that these structures have electronic properties similar to freestanding quantum wells. Accordingly, the band‐edge absorption exhibits excitonic transitions between the lowest 2D subbands of electrons and holes, incrementally shifting to higher energies with decreasing thickness. Due to the degeneracy of the valence band in II–VI compound NPLs, one observes two absorption peaks associated with the lowest subbands of light and heavy holes in these systems,^[^ ^3^, ^11^ ^]^ similar to absorption spectra of epitaxially grown quantum wells. In perovskite NPLs with nondegenerate valence and conduction bands, the absorption shows only one band‐edge excitonic transition.^[^ ^6^, ^12^ ^]^ In both cases, the photoluminescence (PL) comes from the lowest absorption subband and does not display a significant Stokes shift.

Four essential factors influence the outstanding luminescence properties of NPLs: i) An immense exciton binding energy (*E*_*X*_ = 100–500 meV) results from the strong spatial confinement of carriers in the direction perpendicular to the NPL surface.^[^ ^13^ ^]^ The electron–hole Coulomb interaction is significantly enhanced due to the small dielectric constant of the media surrounding the NPLs, which reduces the dielectric screening. Consequently, the effective radius of the 2D exciton, a*, is tiny, and thus the exciton radiative decay rate, proportional to 1/(a*)^2^, is strongly increased. ii) The second factor enhancing the radiative decay rate is the narrow emission linewidth, which results from the low inhomogeneous broadening of the NPL samples due to an absence of a thickness fluctuation of the NPLs. iii) There is a strong coupling of photons to NPL excitons whose transition dipoles are parallel to the NPL surface. This is because dielectric screening does not reduce the magnitude of the photon electric field for polarization parallel to the NPL surface. iv) Finally, at low temperatures, the exciton giant oscillator strength connected with the coherent exciton motion in NPLs leads to a further increase of the radiative recombination rate.^[^ ^14^ ^]^ In CdSe NPLs, the shortening of the radiative decay time^[^ ^3^, ^15^ ^]^ and giant oscillator transition strength^[^ ^16^, ^17^ ^]^ have been reported already, suggesting that all the above‐discussed phenomena could bring the radiative decay time of various NPLs down to the range of tens to a few hundreds of ps.

A severe obstacle potentially prohibiting the realization of such super‐fast emission from NPLs results from the fine structure splitting. The electron–hole exchange interaction is known to split the exciton into a lower energy dark (optically passive) level split from higher‐lying bright (optically active) levels.^[^ ^18^, ^19^, ^20^, ^21^, ^22^ ^]^ A result of this level ordering is that at very low temperatures and assuming a thermalized population distribution only the weakly emitting dark exciton state is occupied, and the NPL decay time increases up to microseconds.^[^ ^22^, ^23^ ^]^ However, in NPLs with a bright–dark splitting Δ*E*_BD_ far smaller than the thermal energy at room temperature *E*_th_ ≈ 26 meV, all states are nearly equally populated after excitation and so, even at room temperature, the dark exciton state decreases the radiative decay rate of the bright exciton, 1/τ_r_, by a factor equal to the ratio of the number of the bright exciton states to the number of total exciton states. For halide perovskites this amounts to $k_{r}=\frac{3}{4}\tau_{r}^{-1}$ and for II–VI compounds to $k_{r}=\frac{1}{2}\tau_{r}^{-1}$. Exciton fine‐structure splitting controlled by the electron–hole exchange interaction is enhanced in nanostructures due to spatial and dielectric confinement. Accordingly, in CdSe NPLs Δ*E*_BD_ was measured to be thickness‐dependent and to vary from 2 to 6 meV.^[^ ^22^ ^]^ The splitting decreases the decay rate at room temperature by a factor of 1/2 because the excitons in the dark state are thermally excited into the bright exciton state for *T* > 70 K. This is in contrast to CdSe quantum dots, where Δ*E*_BD_ is on the order of 20 meV, and depopulation of the bright exciton state affects the radiative decay time even at room temperature.^[^ ^24^ ^]^

The surprising decrease of the radiative decay time with temperature previously observed in lead halide perovskite nanocrystals (NCs) led to the suggestion that these nanostructures exhibit a bright–dark exciton inversion on the order of 1 meV due to the Rashba effect.^[^ ^10^, ^25^ ^]^ Still, the exact origin and nature of the Rashba effect remain unclear. Within the Rashba hypothesis, one can use the fine structure splitting of ≈1 meV to estimate the magnitude of the Rashba terms, which amount to 0.3–0.45 eV·Å. The Rashba effect can only occur in the absence of inversion symmetry. In NPLs, this symmetry breaking could be induced by surface reconstructions or by the surface ligands used to passivate the NPLs.^[^ ^26^ ^]^ Studies of CsPbBr_3_ NPLs show, however, that the dark‐bright splitting of the exciton is surprisingly large, ≈22 meV in 2ML thick NPLs.^[^ ^27^ ^]^ This is significantly larger than the Rashba‐induced effective exchange^[^ ^28^, ^29^ ^]^ and thus would likely negate the Rashba inversion if this were present. Accordingly, it is vital to precisely determine and verify the magnitude of the exchange splitting dependent on the anisotropy of the NPLs both theoretically and experimentally.

In this article, we show that PL spectra and time‐resolved (TR‐)PL decays of 2–8 ML thick Cs_*n* − 1_Pb_*n*_Br_3*n* + 1_ NPLs in the 4–100 K temperature range are in excellent agreement with effective mass modeling of the 2D exciton fine structure taking into account only the electron–hole exchange interaction. Consequently, these results do not necessitate the inclusion of a Rashba effect for their interpretation. We find a thickness‐dependent bright–dark exciton splitting Δ*E*_BD_ reaching up to 32.3 meV for the 2 ML thick NPLs, constituting the largest ever reported value.^[^ ^27^, ^30^ ^]^ Our effective mass model successfully describes the experimental thickness dependence using the long‐ and short‐range (LR and SR) exchange interaction. Taking into account the anisotropy of the band‐edge Bloch functions induced by strong, 1D confinement in the NPLs, we find the spatial and dielectric confinement to significantly enhance the interaction. Moreover, the model reveals a large splitting of the bright exciton states into a component with the transition dipole polarized in the plane of the NPLs and a component with the transition dipole oriented perpendicular to the NPL surface. Interestingly, we predict an inversion of the bright levels for the thinnest (2ML) NPLs, where the out‐of‐plane dipole becomes energetically favorable.

The bright exciton splitting is between 5 and 16 meV for the 2–8 ML NPLs, indicating that the bright levels cannot be approximated as a single state. We confirm this finding through TR‐PL measurements, which show that a two‐level bright–dark exciton model cannot accurately reproduce the observed lifetime dependence on temperature and NPL thickness. Using the splittings calculated from the effective mass model, we employ a novel three‐level model with two bright states split into in‐plane and out‐of‐plane components energetically above a lower‐lying dark state. We obtain good model agreement with the measured PL decay rates for all thicknesses and temperatures. Importantly, our findings and model are not only valid for halide perovskites but can be easily generalized to other semiconductor systems that are well described by effective mass theory. Accordingly, the model should provide a valuable template for other excitonic systems, especially those exhibiting strong quantum confinement.

## 2 Results

**Figure 1:**
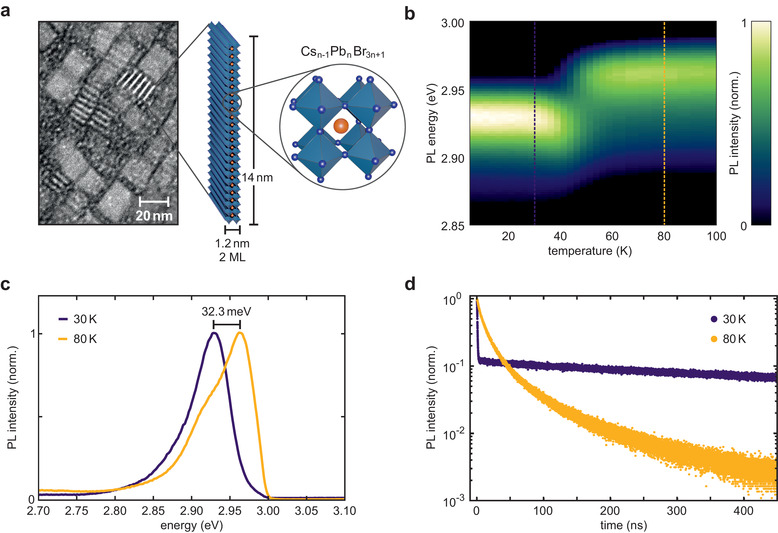
Temperature‐dependent and TR‐PL spectroscopy on 2 ML CsPb_2_Br_7_ NPL thin films. a) TEM image of the 2 ML sample and a scheme depicting the structure of a 2 ML NPL and its atomic crystal structure. b) Normalized, temperature‐dependent PL spectra of a 2 ML NPL thin film. The emission maximum undergoes a sharp 32.3 meV large blueshift at 50 K. c) Line traces taken from (b) at 30 (dark) and 80 K (yellow) show the blueshift prominently. The spectrum at 80 K appears to comprise two merged peaks. d) TR‐PL decay at the respective emission maxima acquired at 30 and 80 K. The low temperature decay exhibits two vastly different lifetimes, a fast one of τ_fast_ < 600 ps (faster than our IRF) and a slow component with τ_slow_ > 1 µs indicative of a bright exciton level above a dark exciton level.

The perovskite NCs used for this study were 2D NPLs with the chemical composition Cs_*n* − 1_Pb_*n*_Br_3*n* + 1_. The thickness of these NPLs assumes discrete values, which we denote as the number of monolayers, *n*, of the NPL. Each ML corresponds to the height of a [PbBr6]^4 −^ octahedron, ≈5.9 Å (**Figure** **1**a).^[^ ^31^ ^]^ Our synthesis of the NPLs is based on a slightly modified version of a previously reported synthesis^[^ ^32^ ^]^ (see Experimental Section). The resulting NPLs not only have higher quantum yields than previously reported, reaching up to 80 %, they are also extremely stable. In dispersion, they remain unchanged for a few weeks and even deposited onto substrates without further encapsulation, they remain stable for at least a week. This allows us to carry out detailed morphological characterization and spectroscopy on thin films and even individual NPLs. The as‐synthesized NPLs have between two and eight MLs with corresponding thickness ranging from 1.2 to 4.7 nm. Importantly, each synthesis yields almost exclusively NPLs of only one thickness, as determined by linear optical spectroscopy. The NPLs are square‐shaped with lateral dimensions of 14 ± 2 nm, as observed by scanning transmission electron microscopy in high‐angle annular dark‐field mode (STEM‐HAADF) and shown here for the case of 2 ML NPLs (Figure 1a; see Figure S1 and Table S1, Supporting Information for other thicknesses). Oleylamine and oleic acid ligands passivate the NPLs to enhance their optical properties and stabilize them against fusion with other NPLs. As previously observed, the organic ligand shell leads to a minimum spacing of 2.5 nm between the perovskite layers, which can be seen particularly in thin films.^[^ ^33^ ^]^ The strong confinement of the NPLs in 1D leads to a significant shift of the absorption onsets and PL emission peaks from the bulk value of 2.36^[^ ^34^ ^]^ up to 2.85 eV for the 2 MLs when dispersed in hexane (see Figure S2, Supporting Information).

Using a home‐built µ‐PL setup with excitation provided by a ps‐pulsed white light laser and operating at temperatures down to 4 K, we can investigate the temperature dependence of the NPLs' PL. For this, we drop‐cast the NPL dispersions onto SiO_2_‐coated Si substrates. There is a notable shift of the PL as the temperature is decreased from room temperature to 4 K due to thermal lattice expansion and exciton‐phonon coupling.^[^ ^35^, ^36^ ^]^ Interestingly, in the 2 ML sample, there is a sharp jump of 32 meV in the emission wavelength at 50 K, from 2.96 eV down to 2.93 eV (Figure 1b). The jump is also present in the spectra of NPLs of other thicknesses. However, the size of the jump and the temperature at which it occurs decrease with increasing NPL thickness, *d* (see Figures S3 and S4, Supporting Information). Comparing the spectra at temperatures above and below this jump, we conclude that the emission stems from two separate energetic states rather than a gradual shift (Figure 1c). This is confirmed by fitting the spectra with two Gaussians fixed in energetic position but with varying intensities (see Figure S5, Supporting Information). CsPbBr_3_, both in bulk and in cube‐shaped NCs, assumes its lowest energy orthorhombic phase already at room temperature.^[^ ^37^, ^38^ ^]^ XRD results (data not shown) support this to hold for the NPLs, consequently, a phase‐change cannot explain the observed jumps. Temperature‐dependent TR‐PL helps to shed light on the nature of these two states (Figure 1d). At temperatures above the jump, the PL decay can be reproduced well with a single exponential and a lifetime on the order of 10 ns. This lifetime becomes longer as the temperature is reduced. At a certain low temperature, the decay becomes bimodal with a short component below the instrument response function of our system of 600 ps and a very long component on the order of 1 µs. The amplitude of the fast component increases rapidly as the temperature is further reduced and then remains constant for the lowest temperatures. These results indicate that the observed PL properties are a result of bright and dark exciton state emission.^[^ ^22^, ^27^ ^]^ Accordingly, the PL jump observed above is due to a splitting of the dark and bright exciton: Δ*E*_BD_ = 32.3 meV. This constitutes the largest such splitting in any semiconductor system reported to date, being nearly six times as large as in CdSe NPLs of comparable thickness.^[^ ^22^ ^]^ This further supports the theoretical work stating that any possible energy level inversion induced by a Rashba effect would be negated by an enhanced electron–hole exchange interaction in strongly confined perovskite NCs.^[^ ^29^, ^30^ ^]^

A two‐level kinetic model, with a dark exciton and a slightly higher‐lying bright exciton level, is typically employed to describe the excited states of the system.^[^ ^27^ ^]^ Excitons in each excited state can relax down to the ground state, or they can transition between the two excited states. Applying this model to the TR‐PL data, we find that the fit can reproduce the observed lifetimes. However, the fine‐structure splitting values obtained do not match the experimentally deduced values (see Figure S6, Supporting Information). The bright exciton level is a triplet state, which is degenerate in the case of cubic crystal structure and isotropic crystal shape.^[^ ^25^, ^29^, ^39^ ^]^ In CsPbBr_3_ NCs with orthorhombic crystal structure and noncubic shape, the degeneracy is lifted and the bright exciton states split in energy. The level splitting was typically observed to be less than 2 meV. In NPLs, however, the shape anisotropy leads to a much larger splitting between the bright excitons with in‐plane (*X* and *Y*) and out‐of‐plane (Z) transition dipoles, analogous to the aforementioned increased bright–dark exciton splitting. Inevitably, the bright triplet state can no longer be approximated by a single state. This in turn must induce a more complex behavior of the exciton dynamics.

### 2.1 2D Exciton in Perovskite Nanoplatelets

**Figure 2:**
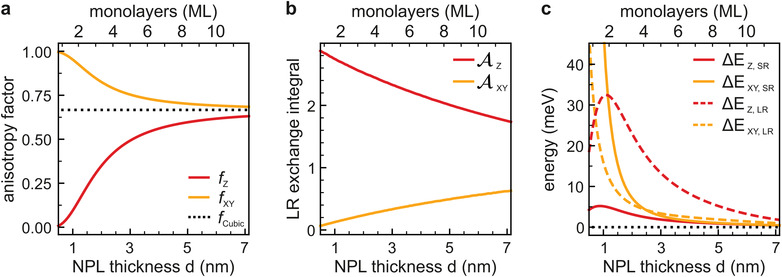
Important quantities calculated within the theoretical effective‐mass model as a function of the NPL thickness. a) Fine structure anisotropy factors $f_{X_{i}}$ due to the anisotropic confinement of band‐edge Bloch functions in a square‐shaped NPL. Out‐of‐plane *f*_*Z*_ is shown in red and in‐plane *f*_*XY*_ is in yellow. The value for an isotropic, cubic NC is 2/3 as depicted by the dashed black line. b) Dimensionless LR exchange integrals given by Equation (S25), Supporting Information. c) LR (dashed lines) and SR (solid lines) exchange contributions to the splitting between the dark exciton and out‐of‐plane bright exciton *Z* (red) and between the dark exciton and in‐plane bright exciton *XY* (yellow).

To explain the experimentally observed, unusually large bright–dark splitting and bright sublevel splitting, we have developed the effective‐mass theory of excitons in 2D perovskite NPLs. As discussed in the introduction, both spatial confinement and dielectric confinement enhance the electron–hole Coulomb interaction and accordingly both the SR and LR electron–hole exchange interaction. The effective exciton radius in these structures is much smaller than the lateral size of the NPLs. This allows for a separation of variables into independent center‐of‐mass $R=\left(m_{e}r_{e}+m_{h}r_{h}\right)/\left(m_{e}+m_{h}\right)$ and relative ***r*** = ***r***_*e*_ − ***r***_*h*_ coordinates.^[^ ^29^, ^40^, ^41^ ^]^ The Hamiltonian is divided into two terms accordingly

(1) $${\hat{H}}_{0}={\hat{H}}_{0,\mathrm{COM}}+{\hat{H}}_{0,\mathrm{rel}}=\frac{{\hat{P}}^{2}}{2M}+\left[\frac{{\hat{p}}^{2}}{2\mu }+V\left(r\right)\right]\;$$

where we employ the exciton translational mass *M* = *m*_*e*_ + *m*_*h*_, the reduced mass *μ* = (1/*m*_*e*_ + 1/*m*_*h*_)^−1^ as well as the center‐of‐mass momentum $\hat{P}={\hat{p}}_{e}+{\hat{p}}_{h}$ and relative momentum $\hat{p}=\left(m_{h}{\hat{p}}_{e}-m_{e}{\hat{p}}_{h}\right)/M$. The Coulomb potential V(r) describing the electron–hole interaction takes into account the difference in dielectric constants of the semiconductor NPL and the surrounding media^[^ ^42^, ^43^ ^]^ and is described using the Hanamura potential (see Section S1, Supporting Information).^[^ ^44^, ^45^ ^]^ The wavefunction of the exciton ground state in an infinite 2D semiconductor layer with thickness *d* in the *z*‐direction is made up of the band‐edge Bloch functions $u_{j_{e}}\left(r_{e}\right)$ and $u_{j_{h}}\left(r_{h}\right)$, the wavefunctions $\sqrt{2/d}\cos\left(\pi z_{e}/d\right)$ and $\sqrt{2/d}\cos\left(\pi z_{h}/d\right)$ that describe electron and hole confinement in the layer respectively, and the relative motion wavefunction $\phi (r_{e}-r_{h})$ with zero angular momentum in the *z* direction. The wavefunction $\phi (r_{e}-r_{h})$ cannot be found analytically, so we obtain it variationally using a 2D “hydrogenic” ansatz wavefunction^[^ ^46^ ^]^

(2) $$\phi_{d}\left(r\right)=\frac{4}{a^{*}\left(d\right)}\frac{1}{\sqrt{2\pi }}\exp\left(-\frac{2|r|}{a^{*}\left(d\right)}\right)\;$$

where a*(*d*), an effective Bohr radius of the 2D exciton, is found using the variational procedure and is strongly thickness dependent (**Figure** **2**a). The determined binding energies (see Figure S7, Supporting Information) match experimentally determined values for a variety of thicknesses.^[^ ^32^, ^47^, ^48^ ^]^

The finite size of the NPL affects the exciton center‐of‐mass motion. Assuming that the edges of a NPL are impenetrable barriers for the exciton, we can write the exciton center‐of‐mass wavefunction as: $2/\sqrt{L_{x}L_{y}}\cos\left(\pi X/L_{x}\right)\cos\left(\pi Y/L_{y}\right)$ for rectangular NPLs with edge lengths *L*_*x*_ and *L*_*y*_. As a result, the total exciton wavefunction in a NPL can be expressed as

(3) $$\begin{array}{ccc} \Psi_{j_{e},j_{h}}^{d}\left(r_{e},r_{h}\right) & = & \frac{4u_{j_{e}}\left(r_{e}\right)u_{j_{h}}\left(r_{h}\right)}{d\sqrt{L_{x}L_{y}}}\cos\left(\frac{\pi X}{L_{x}}\right)\cos\left(\frac{\pi Y}{L_{y}}\right)\cos\left(\frac{\pi z_{e}}{d}\right)\\ & & \cos\left(\frac{\pi z_{h}}{d}\right)\phi_{d}\left(r_{e}-r_{h}\right) \end{array}$$

### 2.2 Bloch Function Anisotropy

In Equation (3), strong spatial confinement in the NPL significantly modifies the conduction band Bloch functions, $u_{j_{e}}\left(r_{e}\right)$, compared to their bulk form for cubic symmetry shown in ref. [25]. Assuming that the perovskite layer of the NPL with an underlying cubic crystal symmetry is surrounded by an infinite confinement potential, we use a six‐band Luttinger Hamiltonian to describe the band‐edge Bloch functions, as detailed in the Experimental Section. The spatial confinement splits the upper *J* = 3/2 fourfold degenerate heavy/light electron states into two subbands similar to the effect of a tetragonal crystal field. We show that the anisotropic part of the Hamiltonian can be expressed as

(4) $$\begin{array}{c} {\tilde{H}}_{2}^{S}\left(0\right)=\left(\begin{array}{cccccc} \Delta_{\mathrm{SO}}+\frac{\delta }{3} & 0 & 0 & 0 & 0 & 0\\ 0 & \Delta_{\mathrm{SO}}-\frac{\delta }{3} & 0 & 0 & \frac{\sqrt{2}\delta }{3} & 0\\ 0 & 0 & \Delta_{\mathrm{SO}}-\frac{\delta }{3} & 0 & 0 & -\frac{\sqrt{2}\delta }{3}\\ 0 & 0 & 0 & \Delta_{\mathrm{SO}}+\frac{\delta }{3} & 0 & 0\\ 0 & \frac{\sqrt{2}\delta }{3} & 0 & 0 & 0 & 0\\ 0 & 0 & -\frac{\sqrt{2}\delta }{3} & 0 & 0 & 0 \end{array}\right)\; \end{array}$$

where Δ_SO_ is the spin‐orbit splitting between the *J* = 3/2 (light electron, heavy electron) and the *J* = 1/2 states (split‐off electron) in the conduction band. The parameter δ can be considered as an effective tetragonal crystal field and is given by

(5) $$\begin{array}{c} \delta =-3\frac{\hbar^{2}}{2m_{0}}\gamma_{2}\frac{2\pi^{2}}{d^{2}}\; \end{array}$$

where $\gamma_{2}=E_{p}/6E_{g}^{\prime }$ is the second Luttinger parameter, $E_{g}^{\prime }$ is the band gap of the NPL (see Section S2, Supporting Information), and *E*_p_ is the Kane energy parameter (see **Table** **1**). These parameters induce a splitting of the conduction band into three bands, analogously to the splitting of the valence band in traditional semiconductors such as GaAs. The lowest‐lying conduction band, also referred to as the split‐off band, has an energy of^[^ ^29^ ^]^

(6) $$\begin{array}{c} E_{c}=\frac{3\Delta_{\mathrm{SO}}-\delta }{6}-\frac{1}{2}\sqrt{\Delta_{SO}^{2}-\frac{2}{3}\Delta_{SO}\;\delta +\delta^{2}} \end{array}$$

and the resulting Bloch functions can be written as^[^ ^29^, ^53^ ^]^

(7) $$\begin{array}{ccc} & & u_{c,1/2}=-\sin\theta Z↑-\cos\theta \frac{X+iY}{\sqrt{2}}↓,\\ & & u_{c,-1/2}=-\cos\theta \frac{X-iY}{\sqrt{2}}↑+\sin\theta Z↓ \end{array}$$

In these expressions, the angle θ is given by^[^ ^53^ ^]^

(8) $$\begin{array}{c} \tan2\theta =\frac{2\sqrt{2}\Delta_{SO}}{\Delta_{SO}-3\delta }\;,\;\left(\theta \leq \frac{\pi }{2}\right)\; \end{array}$$

**Table 1:** CsPbBr_3_ material parameters used for calculation of the level splitting. Parameters are taken from the literature as indicated. The ligands bonded to the NC surface make up the exterior dielectric, so we take ϵ_*o*_ to be the square of the refractive index of oleic acid. The short‐range exchange constant *C* ^SR^ is calculated based on experiments in FAPbBr_3_ ^[^ ^49^ ^]^ together with Ω and *a*_*x*_, using the methodology in ref. [29]. Calculated values of *C* ^SR^ in CsPbBr_3_ and FAPbBr_3_ are similar ^[^ ^29^ ^]^ but larger than the experimental value for FAPbBr_3_, so we use the experimental value in this work

Parameter	Value	Description	Source
*ℏω*_LT_^bulk^	5.4 meV	LT exciton splitting	Experiment^[^ ^50^ ^]^
*E*_p_	27.8 eV	Kane energy	Derived from ℏω_LT_^[^ ^29^, ^50^ ^]^
*a*_*x*_	3.1 nm	Bulk exciton Bohr radius	Theory^[^ ^28^ ^]^
ϵ_*i*_	7.3	Interior dielectric constant	Experiment^[^ ^51^ ^]^
ϵ_*o*_	2.1263	Exterior dielectric constant	Experiment^[^ ^52^ ^]^
ϵ_∞_	4.76	High‐frequency dielectric constant	Derived from *ℏω*_LT_^bulk^ and *E*_p_^[^ ^28^, ^50^ ^]^
μ	0.126 *m*_0_	Reduced effective mass	Experiment^[^ ^51^ ^]^
*m*_*e*_	0.25 *m*_0_	Electron effective mass	Theory^[^ ^28^ ^]^
*m*_*h*_	0.25 *m*_0_	Hole effective mass	Theory^[^ ^28^ ^]^
*E*_g_	2.342 eV	Band gap	Experiment (*T* = 4.2 K)^[^ ^51^ ^]^
Δ_SO_	1.5 eV	Spin‐orbit splitting	Theory^[^ ^29^ ^]^
Ω	0.2104 nm^3^	Pseudocubic unit‐cell volume	Experiment^[^ ^29^ ^]^
*C* ^SR^	315.5 meV	Short‐range exchange constant	Derived from experiment^[^ ^29^, ^49^ ^]^

### 2.3 Fine Structure of the Ground Exciton State

The LR and SR exchange interaction between electron and hole spin states split the fourfold degenerate ground exciton levels in perovskite NPLs described by the wavefunction in Equation (3) into three optically active bright exciton states and one optically passive dark exciton state. The exciton fine structure is typically calculated by splitting the interaction into SR and LR components: Δ*E*_exchange_ = Δ*E*_SR_ + Δ*E*_LR_. We shall initially consider the SR exchange interaction.

#### 2.3.1 Short‐Range Electron–Hole Exchange Interaction

The short‐range exchange interaction has the form of a contact interaction with an effective spin operator acting on the electron and hole Bloch functions^[^ ^29^, ^54^ ^]^

(9) $$\begin{array}{c} {\hat{H}}_{\mathrm{exch}}^{\mathrm{SR}}=\frac{1}{2}C^{\mathrm{SR}}\Omega \left[I-(\sigma_{e}\cdot \sigma_{h})\right]\delta \left(r_{e}-r_{h}\right)\; \end{array}$$

In this expression, *C* ^SR^ is the exchange constant, Ω is the volume of the crystal unit cell, I is the 4×4 unit matrix and $\sigma_{e,h}$ are the Pauli operators acting on the electron and hole spins.

To find the dark–bright exciton splitting one can directly rewrite Equation (9) in the matrix representation using four pairs of electron and hole Bloch functions and diagonalize the matrix. The Bloch functions of the conduction band *u*_c, ±1/2_ are defined in Equation (7) and the Bloch functions of the *s*‐like valence band are *u*_*v*, 1/2_ = |*S*↑〉 and *u*_*v*, −1/2_ = |*S*↓〉. It is more convenient, however, to transform the pair Bloch function basis to the basis of linear dipoles.^[^ ^41^ ^]^ Herein, the basis functions |*X*_*i*_〉 run over |*D*〉, |*X*〉, |*Y*〉, |*Z*〉, which represent the wavefunctions of the dark state and the three bright excitons with transition dipoles aligned along the *X*, *Y*, *Z* directions.^[^ ^41^ ^]^

As we show in Section S3, Supporting Information, the SR exchange Hamiltonian in this basis can be expressed as

(10) $$\begin{array}{c} {\tilde{H}}_{\mathrm{exch}}^{\mathrm{SR}}=C^{\mathrm{SR}}\Theta \left(\begin{array}{cccc} 0 & 0 & 0 & 0\\ 0 & \cos^{2}\theta & 0 & 0\\ 0 & 0 & \cos^{2}\theta & 0\\ 0 & 0 & 0 & 2\sin^{2}\theta \end{array}\right)\; \end{array}$$

where θ is defined in Equation (8) and the overlap factor Θ represents the probability that the electron and hole reside in the same unit cell.^[^ ^29^ ^]^ For the quasi‐2D exciton with envelope wavefunction $f\left(r_{e},r_{h}\right)=\frac{2}{\sqrt{L_{x}L_{y}}}\cos\left(\frac{\pi X}{L_{x}}\right)\cos\left(\frac{\pi Y}{L_{y}}\right)\frac{2}{d}\cos\left(\frac{\pi z_{e}}{d}\right)\cos\left(\frac{\pi z_{h}}{d}\right)\phi_{d}\left(r_{e}-r_{h}\right)$ (see Equation (3)) this factor is given by the expression

(11) $$\begin{array}{c} \Theta =\Omega \;\underset{V}{\overset{}{\;\int \int \;}}d^{3}r_{e}d^{3}r_{h}f^{*}\left(r_{e},r_{h}\right)\delta \left(r_{e}-r_{h}\right)f\left(r_{e},r_{h}\right)=\frac{3}{2d}\Omega |\phi_{d}\left(0\right){|}^{2}\; \end{array}$$

The splitting introduced by the SR exchange interaction between the dark exciton and bright excitons with two degenerate *X* and *Y* dipoles can be written accordingly

(12) $$\Delta E_{X,\mathrm{SR}}=\Delta E_{Y,\mathrm{SR}}=f_{X}C^{\mathrm{SR}}\Omega \frac{3}{2d}{\left|\phi_{d}\left(0\right)\right|}^{2}\;$$

and between the dark exciton and the bright *Z* dipole

(13) $$\Delta E_{Z,\mathrm{SR}}=f_{Z}C^{\mathrm{SR}}\Omega \frac{3}{2d}{\left|\phi_{d}\left(0\right)\right|}^{2}\;$$

where *f*_*Z*_ = 2sin ^2^(θ) and *f*_*X*_ = *f*_*Y*_ = cos ^2^(θ). The difference between Equations (12) and (13) describes the splitting between the bright excitons in the square NPLs for the states with dipoles in‐plane (*X*, *Y*) and out‐of‐plane (*Z*).

#### 2.3.2 Long‐Range Electron–Hole Exchange Interaction

Next, we can consider the LR exchange interaction, which in bulk semiconductors induces a splitting between the longitudinal and transverse (LT) optically active exciton states. To calculate the LR exchange interaction in a NPL, we use the straightforward formalism developed by Cho.^[^ ^55^ ^]^ Cho's analysis begins with the observation that any exciton state is accompanied by a transition dipole density P(r), which is induced by a charge density defined as ρ(r)=−∇·P(r). Cho showed that in this case the LR exchange can be expressed in terms of the Coulomb interaction of this charge density associated with a given exciton state^[^ ^55^ ^]^

(14) $$\begin{array}{c} H_{X_{i}}^{\mathrm{LR}}=\int_{V_{1}}dV_{1}\int_{V_{2}}dV_{2}{\left[-\nabla_{1}\cdot P_{X_{i}}\left(r_{1}\right)\right]}^{*}\frac{1}{\varepsilon_{\infty }\left|r_{1}-r_{2}\right|}\left[-\nabla_{2}\cdot P_{X_{i}}\left(r_{2}\right)\right] \end{array}$$

where ϵ_∞_ is the high frequency dielectric constant that screens the Coulomb interaction.

Using the linear dipole exciton Bloch function basis we identify the polarization P as the transition dipole moment density associated with a given exciton state *X*_*i*_, whose wavefunction is described as $f\left(r_{e},r_{h}\right)\left|X_{i}\right\rangle$. For such a transition the polarization can be written as

(15) $$\begin{array}{c} P_{X_{i}}\left(r_{e}\right)=i\frac{\hbar e}{m_{0}\hbar \omega }\underset{}{\int^{}}d^{3}r_{h}f\left(r_{e},r_{h}\right)\;p_{X_{i}}\delta \left(r_{e}-r_{h}\right)=i\frac{\hbar e}{m_{0}\hbar \omega }f\left(r_{e},r_{e}\right)\;p_{X_{i}} \end{array}$$

where we define the transition energy *E*_2_ − *E*_1_ = ℏω (see Figure S8, Supporting Information) and $p_{X_{i}}$ is the matrix element of the momentum operator taken between the Bloch functions of the conduction and valence bands for the *X*_*i*_ exciton state. Previously, we successfully used this procedure to calculate the LR exchange interaction in quasicubic perovskite NCs^[^ ^28^, ^29^ ^]^ and perovskite nanowires.^[^ ^56^ ^]^ In Section S4, Supporting Information, we apply the same procedure to square‐shaped NPLs, with dimensions *L* × *L* × *d*. Straightforward calculations yield the following expression for the dark‐bright exciton splitting for the exciton states *X*_*i*_

(16) $$\Delta E_{X_{i},\mathrm{LR}}\left(d\right)=\frac{\hbar \omega_{\mathrm{LT}}\left(d\right)}{2}f_{X_{i}}A_{X_{i}}\left(d/L\right)\left(\frac{\Theta }{\Theta_{\mathrm{bulk}}}\right)$$

where $\hbar \omega_{\mathrm{LT}}\left(d\right)=\hbar \omega_{\mathrm{LT}}^{\mathrm{bulk}}{\left(E_{g}/\hbar \omega \right)}^{2}$ is the *d*‐dependent LT splitting energy. The bulk exchange overlap $\Theta_{\mathrm{bulk}}=\Omega /\left(\pi a_{x}^{3}\right)$ is determined by the bulk exciton radius *a*_*x*_. $A_{X_{i}}\left(d/L\right)$ are dimensionless integrals defined in Equation (S25), Supporting Information that depend on the ratio of the NPL thickness to their lateral size, *d*/*L*. The dependence of $A_{X}$ and $A_{Z}$ on the NPL thickness is calculated numerically and shown in Figure 2b and Figure S9, Supporting Information. One can see that as *d*/*L* → 0, $A_{X}\to 0$ and $A_{Z}\to 3$. In square‐shaped NPLs the *XY* dipole states remain degenerate, while the LR exchange interaction splits these states for the case *L*_*x*_ ≠ *L*_*y*_.

Combining the SR and LR exchange interaction, we obtain the total splitting of the dark state to the bright *Z* and (*X*, *Y*) states in a square NPL

(17) $$\begin{array}{ccc} \Delta E_{Z} & = & \frac{3f_{Z}}{2d}{\left|\phi_{d}\left(0\right)\right|}^{2}\left[C^{\mathrm{SR}}\Omega +\frac{\hbar \omega_{\mathrm{LT}}\left(d\right)}{2}\pi a_{x}^{3}A_{Z}\left(d/L\right)\right]\\ \Delta E_{X} & = & \Delta E_{Y}=\frac{3f_{X}}{2d}{\left|\phi_{d}\left(0\right)\right|}^{2}\left[C^{\mathrm{SR}}\Omega +\frac{\hbar \omega_{\mathrm{LT}}\left(d\right)}{2}\pi a_{x}^{3}A_{X}\left(d/L\right)\right] \end{array}$$

**Figure 3:**
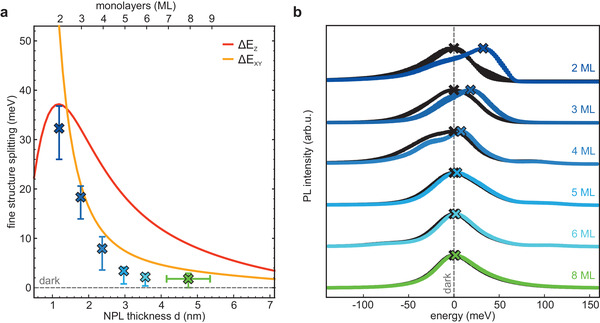
Thickness dependence of the bright–dark exciton level splitting in NPLs. a) Total exchange splitting (SR and LR) for the bright out‐of‐plane *Z* (red line) and in‐plane (*X*, *Y*) (orange line) exciton states from the dark state in a square NPL with lateral side length *L* = 14 nm. The dark exciton level is the lowest for all thicknesses. The bright *Z* level lies above the *XY* level for all thicknesses above the 2 ML NPLs. Splitting energies determined experimentally from the shift of the PL spectra, as depicted in (b). Horizontal error bars on the 8 ML sample indicate a possible minor contribution from 7 ML and 9 ML NPLs. The *y*‐error bars denote the standard deviation of the PL maxima analyzed in (b) to obtain the energetic position of the exciton levels. b) Temperature‐dependent PL spectra of NPL thin films. For each thickness, several spectra below the jump (black dots) and above the jump (colored dots) are superimposed to help determine the location of the dark and lowest bright exciton states, denoted by the crosses. Temperature intervals in which the PL jump occurs are shown in Table S2, Supporting Information. All spectra are offset horizontally according to the respective energetic position of the dark exciton level of each NPL. Accordingly, the PL jump becomes larger as the thickness of the NPL decreases, from 1.8 meV for 8 MLs up to 32.3 meV for 2 MLs.

The calculated dependence of these energies on Cs_*n* − 1_Pb_*n*_Br_3*n* + 1_ NPL thickness is plotted in **Figure** **3**a (solid lines), where the dark exciton level is set to Δ*E* = 0. The material parameters used for this calculation are given in Table 1. Importantly, the bright excitons are always located energetically above the dark exciton state. The previously reported bright–dark level inversion in CsPbBr_3_ NCs was due to the Rashba effect,^[^ ^25^ ^]^ and large Rashba terms can have a dramatic impact on exciton fine structure.^[^ ^57^ ^]^ However, the Rashba terms in CsPbBr_3_ are moderate, and the magnitude of the exchange interaction and the crystal structure anisotropy in CsPbBr_3_ NCs are significantly smaller than the corresponding effects in the NPLs. Hence, for the NPLs the addition of possible Rashba terms would not affect the ordering of dark and bright excitons and are not considered here. For nearly all NPL thicknesses, the in‐plane bright states *X*, *Y* are energetically lower than the out‐of‐plane bright states *Z*, as one would intuitively expect. Interestingly, there is a crossing of these states at *d* ⩽ 1.4 nm, below which the exciton state with out‐of‐plane transition dipole is the energetically favorable one. This *d* value lies between the 2 and 3 ML NPLs' thickness. Accordingly, the polarization of PL emitted from the lowest bright exciton state should be parallel to the NPL surface for all NPLs except for the thinnest 2 ML NPLs, for which the PL should be polarized perpendicular to the NPL surface. Furthermore, the calculations show that the total splitting between (*X*, *Y*) and *Z* bright excitons exhibits a prominent thickness dependence. Increasing initially from 8 to 13.2 meV from 2 to 4 MLs, the splitting decreases to 4.5 meV for the 8 ML NPLs. Accordingly, one cannot assume the bright states to act as one degenerate level, revealing why the two‐level model could not explain the TR‐PL data.

### 2.4 Spectroscopically Determined Bright–Dark Splitting

Having determined the thickness‐dependent bright–dark level splitting for the NPLs, we can revisit the PL spectra of the ensembles (Figure 3b). As mentioned earlier, all samples exhibit a jump in the PL maximum at a given temperature. The jump is very prominent in the 2 ML sample and can easily also be observed in the 3 ML and 4 ML samples. The small shoulder on the low energy side of the 4 ML spectra is due to a small sample inhomogeneity, likely due to varying lateral sizes. This, however does not affect the analysis here. For the thicker NPLs, the PL jump becomes more of a gradual shift and is more difficult to discern by eye (see Figure S10, Supporting Information). To quantify the jump, we must first consider the gradual PL shift arising from the combination of exciton–phonon coupling and lattice expansion, also referred to as bandgap renormalization.^[^ ^35^, ^58^ ^]^ These effects are strongly thickness and temperature‐dependent. We determine their contribution to the overall PL shift for the 2–5 ML NPLs and for weakly‐confined nanocubes as an upper limit in the temperature ranges around the jump (see Table S2, Supporting Information).^[^ ^36^ ^]^ The total shift decreases from 3.6 meV for the 2 ML NPLs to 1.7 meV for the 5 ML NPLs and 1.0 meV for the nanocubes. Consequently, for all but the thickest NPLs, this effect plays only a negligible role. Taking this into account, by plotting several spectra just below (black dots) and just above the jump (colored dots), we can more easily determine the spectral positions of the bright and dark states. For each of these ranges the contribution from bandgap renormalization is far below 1 meV. Notably, for each thickness, the temperature ranges are in good agreement with the transition temperatures predicted by the extracted energetic width of the jumps and with the PL decay curves discussed below. The crosses in Figure 3b denote the respective PL maxima, and the spectra are offset according to the spectral position of the dark state. It is evident that the shift between the two maxima, which corresponds to Δ*E*_BD_, increases with diminishing thickness of the NPLs from 1.8 meV for the 8 ML NPLs to 32.3 meV for the 2 ML NPLs. Accordingly, the PL jump can only be easily discerned experimentally for the thinner NPLs and appears as a localized enhanced bandgap renormalization for the thicker NPLs. Thus, the experimental values for the thicker NPLs can be seen as an upper limit for the splitting energy. Importantly, experimental and theoretical splittings are in excellent agreement, especially for the thinnest NPLs. Interestingly, the value for the 2 ML sample lies closer to the curve for the *Z* dipole, supporting the notion of a bright exciton level inversion for the thinnest NPLs. Polarization‐dependent spectroscopy on single NPLs could fully confirm this prediction. For the thicker NPLs, the experimental values lie below the predicted ones. This could be due to the fact that determining the splitting becomes progressively more difficult as the splitting is reduced. Also, at low temperatures with a reduced linewidth broadening of the PL spectra, one can see that they are not fully monodisperse and contain a small population of NPLs of different thickness. TEM images also confirm this (see Figure S1, Supporting Information). The slight polydispersity could affect the measurements for the thicker NPLs, where the PL positions do not vary much between NPLs with ΔML = ±1. Another aspect that we have so far disregarded is the fine structure splitting resulting from the crystal structure anisotropy itself. The NPLs assume the orthorhombic crystal structure, which should lead to an additional energy level splitting.^[^ ^9^, ^59^ ^]^ While this is small compared to Δ*E*_BD_ in the thinner NPLs; the same cannot be said for the thicker NPLs. If the crystal structure aligns preferentially along one NPL direction, this could either reduce or increase the overall splitting of the exciton fine structure.

### 2.5 Exciton Lifetimes

**Figure 4:**
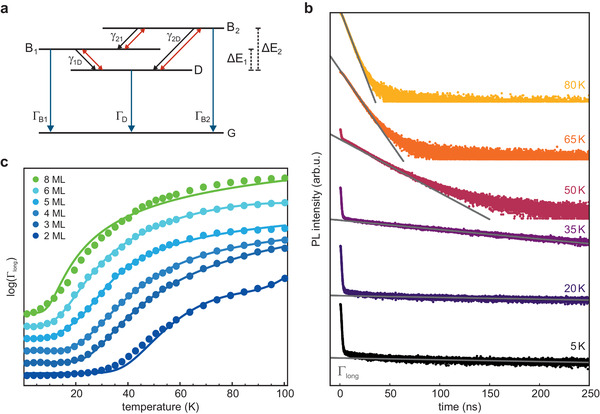
PL decay in the NPLs explained by a three‐level model. a) Three‐level model developed to explain the TR‐PL data. Three excited states (a lowest‐lying dark state, D, and two bright states, *B*_1_ and *B*_2_) can decay directly to the ground‐state, G. Excitons can also exchange between the three excited states via one‐phonon interaction. Bright–dark splitting between the two bright levels is given by Δ*E*_1, 2_. b) Series of temperature‐dependent PL decay traces between 5 and 80 K for 4 ML NPL thin films. The decay rate of the slow component, Γ_long_, which is determined from an exponential fit function, becomes progressively faster as the temperature is increased. c) The slow decay rate Γ_long_ in dependence of temperature for all NPL samples (colored dots, with color coding corresponding to Figure 3). The data points are plotted logarithmically (in ns^−1^) and offset vertically for clarity. All samples show the same trend with a constant decay rate, which rapidly becomes larger starting from a given temperature. This temperature, coinciding with the experimentally observed jump in the PL spectra, decreases with increasing NPL thickness. This is due to a reduced exciton level splitting and an easier thermal activation from lower lying levels. A fit of Γ_long_ employing the three‐level model (solid lines) shows good agreement to the experimental data for all thicknesses.

The PL decay times could not be explained with the commonly used two‐excited‐level model^[^ ^27^ ^]^ as mentioned above. As we showed in the last section, the bright level splitting is between 5–16 meV for the NPLs studied here. Consequently, one should instead consider a three‐level system (see **Figure** **4**a). In this model, the lowest excited state D lies beneath two bright levels denoted *B*_1_ and *B*_2_, as the *X*, *Z* ordering changes between 2 and 3 MLs. Optical excitation occurs far above these levels, hence, excitons will relax quickly, populating all three excited levels. At low temperatures, the bright states will be emptied rapidly, as their recombination rates, $\Gamma_{B_{1}}$ and $\Gamma_{B_{2}}$ are very large. Excitons in the dark state reside there for a long time, since the direct recombination rate Γ_D_ is very low. Nonradiative recombination processes are not included in this model; such processes would act to reduce the overall quantum yield if they were significant. With increasing temperature, a thermally induced (phonon‐mediated) transition between the excited states becomes more likely. Thus, excitons in the dark state can also decay indirectly via thermal excitation into either of the bright states. Accordingly, at the lowest temperatures, the prolonged decay observed in the TR‐PL measurements (denoted Γ_long_) corresponds exactly to Γ_D_. Thus, the rate can be obtained by fitting the long tail (Figure 4b, Figure S11, Supporting Information for full data). At higher temperatures, other pathways are accessible, significantly complicating the population evolution of the dark state (see Section S5, Supporting Information). Consequently, the slow decay observed Γ_long_ is no longer equal to Γ_D_. Nevertheless, we can model this decay rate and compare the calculations to the TR‐PL data using the three‐excited‐level model. To reduce the number of free parameters for fitting, we have fixed the energy levels of the three states and the ratio $\Gamma_{B_{1}}/\Gamma_{B_{2}}$ according to the results obtained from the theoretical model described above. The slow decay rate Γ_long_ was measured for all NPL thicknesses and is plotted for the temperature range 4–100 K (Figure 4c, filled circles; the curves are offset vertically for clarity). At the lowest temperatures, Γ_long_ is constant, but then rapidly increases. The temperature for which this change occurs is strongly dependent on the NPL thickness. While the decay rate remains roughly constant up to 25 K for the 2 ML sample, for the 8 ML sample, there is nearly no constant rate. These results agree with the previously determined thickness‐dependent bright–dark exciton splitting, as thermal excitation from the dark state is activated at lower temperatures for increasing NPL thickness. The fits resulting from the three‐level model (solid lines) accurately reproduce the experimental data for all NPLs, especially for the 3–6 ML NPLs, validating the model. The less‐than‐optimal fitting in the case of the 8 ML sample is likely due to the inhomogeneity of the sample, resulting in different lifetimes and also due to energy transfer via Förster resonance energy transfer occurring.^[^ ^33^ ^]^ In the 2 ML sample, the small mismatch in the range 30–80 K is caused by two effects. First, the emergence of a self‐trapped exciton in this temperature range (see Figure S12, Supporting Information) means there is an additional decay pathway from excitons residing in the dark state.^[^ ^60^ ^]^ The other effect is the effective‐mass model's tendency to slightly overestimate Δ*E*_1, 2_. As the model still has five free parameters, the fit is qualitative rather than quantitative. Yet, it demonstrates that the calculated splitting energies can explain the TR‐PL data. Additional experiments, including the temperature dependence of the fast PL decay rate, are necessary to improve the model to extract meaningful transition rates.

## 3 Discussion

This study demonstrates and quantifies the influence of shape anisotropy on the exciton fine structure in 2D halide perovskite NPLs. For the first time, we show that this anisotropy leads to an energetic splitting of the bright triplet into in‐plane and out‐of‐plane levels. Temperature‐dependent PL‐spectroscopy on 2–8 ML thick NPLs reveals that in all NPLs independent of their thickness the lowest exciton sublevel is an optically forbidden dark exciton. The bright–dark exciton splitting increases from 1.8 meV for the 8 ML up to 32.3 meV for the 2 ML NPLs, constituting the largest value ever measured. Using an effective‐mass model, we calculate the exciton fine structure in the NPLs. The model quantitatively explains this large splitting, showing it is completely described by the electron–hole exchange interaction. This interaction is significantly enhanced by the strong confinement of carriers in the out‐of‐plane direction in the NPLs. Notably, the magnitude of these shifts shows that the bright levels cannot be considered degenerate and must be individually accounted for. Accordingly, we can use the splitting energies and the decay rates obtained from the temperature‐dependent TR‐PL measurements to calculate the expected PL spectra for each NPL sample (see Figure S13, Supporting Information). These PL maps show striking agreement with the experimentally obtained ones (compare Figure S3, Supporting Information) with the slight exception for the 2 ML case. There, a non‐negligible emission from the bright‐states at 0 K is likely due to the aforementioned overestimation of the bright–bright level splitting. Nevertheless, the agreement between the calculations and experiment strongly supports our interpretation of the data and the model of the excitonic fine structure we have developed here.

Interestingly, the model predicts an inversion of the bright exciton states for NPLs thinner than 3 ML. Accordingly, for all NPLs but the 2 MLs, the polarization of the lowest optically allowed transition lies in the NPL plane, while in the 2 MLs it is oriented perpendicular to the NPL plane. Polarization‐dependent spectroscopic studies of single NPLs are needed to confirm this prediction. Using the energy level splitting determined from the model, we can reproduce and explain the temperature dependence of the TR‐PL data for all NPL samples. This fitting supports the three‐level model of the 2D system with a lowest‐lying dark exciton and two bright exciton levels split by several meV. With splitting energies of this magnitude, an impact should still be visible at room temperature, especially for the thinnest NPLs. In two previous studies we had observed that the PL decayed faster in thicker than in thinner NPL.^[^ ^32^, ^33^ ^]^ With a concomitant increase in quantum yield and a reduced nonradiative decay rate, this meant that a larger radiative decay rate must be the cause of the faster PL decay in the thicker NPLs. However, this stood in contrast to the behavior of quantum wells, for which it was deduced that the radiative decay rate should increase as the quantum well is reduced in size.^[^ ^14^ ^]^ The findings in this work can help to explain the apparent contradiction if one considers that even at room temperature an exciton will spend a considerable amount of time in the lowest dark state. Thermal excitation to a bright state (feeding) will lead to a prolonged PL emission from this bright state, masking its actual radiative decay rate.

To describe the experimental results in Cs_*n* − 1_Pb_*n*_Br_3*n* + 1_ NPLs we have developed a model that takes into account the anisotropy of the band‐edge Bloch functions introduced by spatial confinement. Additionally, we establish a tractable and straightforward approach to the calculation of the LR exchange interaction. These effects are essential in our system, and their inclusion will likely be necessary for other systems exhibiting strong shape anisotropy. The model can easily be extended to rectangular NPLs (*L*_*X*_ ≠ *L*_*Y*_, both ≫*d*) by simply replacing *L* in the LR exchange integrals as appropriate. The model is also broadly generalizable to any semiconductor NPL system that is well described by effective mass theory. We believe it will be a valuable template for use in other excitonic systems such as in multilayered 2D lead halide perovskites.^[^ ^60^ ^]^

Further refinement to the model could be provided by incorporating both shape and crystal structure anisotropy. For this, one would need to know whether and how the crystal structure aligns with the NC anisotropy. Polarization‐dependent PL spectroscopy predominantly on single NPLs could help to elucidate this question. Nevertheless, our study provides a novel theoretical model to determine the energy level structure in highly anisotropic nanostructures, essential for optimizing these further and incorporating them into optoelectronic applications.

The level separation for the 2–8 ML NPLs is larger than the Rashba‐induced effective exchange previously predicted in 3D CsPbBr_3_ NCs,^[^ ^29^ ^]^ validating the choice to neglect the Rashba terms in our model, and suggesting that the ground exciton state in CsPbBr_3_ NPLs will remain dark in the absence of factors that substantially enhance the Rashba effect. The search for NPLs with sufficiently strong Rashba interactions to exhibit a bright ground exciton should be continued.

## 4 Experimental Section

### Synthesis

Materials: Cs_2_CO_3_ (cesium carbonate, 99 %), PbBr_2_ (lead(II) bromide, ⩾98 %), oleic acid (technical grade 90 %), oleylamine (technical grade 70 %), acetone (for HPLC, ⩾99.9 %), toluene (for HPLC, ⩾99.9 %), and hexane (for HPLC, ⩾97.0 %, GC) were purchased from Sigma‐Aldrich. All chemicals were used as received.

The PbBr_2_ and Cs precursor solutions, as well as the PL enhancement solution, were prepared according to Bohn et al.,^[^ ^32^ ^]^ the synthesis of the NPLs, however, was modified somewhat.

The synthesis was carried out under ambient atmosphere at room temperature. A vial was charged with PbBr_2_ precursor solution and Cs precursor solution was added under stirring at 1200 rpm. The corresponding volumes for different thicknesses can be found in Table S4, Supporting Information. After 10 s, acetone (2 mL) was added and the reaction mixture was stirred for 1 min. The mixture was centrifuged at 4000 rpm for 3 min and the precipitate was redispersed in hexane (1.8 mL). To enhance the stability and emission properties of the NPLs, an enhancement solution (200 µL) was added.

### Electron Microscopy

STEM‐HAADF mode was performed with a probe‐corrected Titan Themis (FEI) at an acceleration voltage of 300 kV.

### PL Spectroscopy

Steady‐state and TR‐PL measurements were conducted using a pulsed laser (NKT Photonics, SuperK Fianium FIU‐15) and an excitation wavelength of λ_central_ = 410 nm. The laser was operated with a repetition rate of 1.95 MHz and an excitation power of 9 Wcm^−2^. The light was collected with a 50× objective (Mitutoyo, Plan Apo HR 50x) with a working distance of 5.2 mm. Steady‐state PL spectra were measured using a SpectraPro HRS‐500 spectrometer with a 300 mm^−1^ grating and a PIXIS charge‐coupled device (all Teledyne Princeton Instruments). TR‐PL measurements were conducted using a SPAD (Excelitas Technologies, SPCM‐AQRH‐16‐BR1) and a TCSPC‐device (Swabian Instruments, Time Tagger 20). The silicon substrates (300 nm SiO_2_ layer) with the NPLs drop‐casted onto them were cooled using an attoDRY 800 closed‐cycle liquid helium cryostat (Attocube). Room‐temperature PL (and absorption) of the dispersions was measured using a commercial spectrometer (FLUOROMAX‐Plus, HORIBA).

### Bloch Function Anisotropy

The anisotropy factors *f*_*z*_ and *f*_*XY*_ (see Figure 2a) reflect the effect of the anisotropy between the in‐plane and out‐of‐plane directions in a NP on the band‐edge Bloch functions. The Bloch function of the valence band had *s*‐symmetry and can be written as *u*_*v*, 1/2_ = |*S*〉|↑〉 and *u*_*v*, −1/2_ = |*S*〉|↓〉, where the term |*S*〉 denotes an orbital state which transforms as the real spherical harmonic with *l* = 0 while |↑〉, |↓〉 are the usual spin eigenstates.^[^ ^25^ ^]^

For the conduction bands, the Bloch functions were more complex due to the *p*‐symmetry of these bands. The conduction bands of perovskite semiconductors can be described using the six‐band Luttinger Hamiltonian in the spherical approximation. In a basis of Bloch functions |*J*, *J*_*z*_〉 taken in the order |3/2, 3/2〉, |3/2, 1/2〉, |3/2, −1/2〉, |3/2, 3/2〉, |1/2, 1/2〉, and |1/2, −1/2〉, the Hamiltonian has the form^[^ ^61^ ^]^

(18) $$\tilde{H}={\tilde{H}}_{1}+{\tilde{H}}_{2}$$

where

(19) $${\tilde{H}}_{1}=\frac{1}{2m_{0}}\left(\begin{array}{cccccc} \gamma_{1}p^{2} & 0 & 0 & 0 & 0 & 0\\ 0 & \gamma_{1}p^{2} & 0 & 0 & 0 & 0\\ 0 & 0 & \gamma_{1}p^{2} & 0 & 0 & 0\\ 0 & 0 & 0 & \gamma_{1}p^{2} & 0 & 0\\ 0 & 0 & 0 & 0 & \gamma_{1}p^{2} & 0\\ 0 & 0 & 0 & 0 & 0 & \gamma_{1}p^{2} \end{array}\right)$$

with $p^{2}=p_{x}^{2}+p_{y}^{2}+p_{x}^{2}$ and

(20) $${\tilde{H}}_{2}=\frac{1}{2m_{0}}\left(\begin{array}{cccccc} 2m_{0}\Delta_{\mathrm{SO}}+\gamma_{2}p_{\mathrm{eff}}^{2} & -2\sqrt{3}\gamma_{2}p_{-}p_{z} & -\sqrt{3}\gamma_{2}p_{-}^{2} & 0 & \sqrt{6}\gamma_{2}p_{-}p_{z} & \sqrt{6}\gamma_{2}p_{-}^{2}\\ -2\sqrt{3}\gamma_{2}p_{+}p_{z} & 2m_{0}\Delta_{\mathrm{SO}}-\gamma_{2}p_{\mathrm{eff}}^{2} & 0 & -\sqrt{3}\gamma_{2}p_{-}^{2} & \sqrt{2}\gamma_{2}p_{\mathrm{eff}}^{2} & -3\sqrt{2}\gamma_{2}p_{-}p_{z}\\ -\sqrt{3}\gamma_{2}p_{+}^{2} & 0 & 2m_{0}\Delta_{\mathrm{SO}}-\gamma_{2}p_{\mathrm{eff}}^{2} & 2\sqrt{3}\gamma_{2}p_{-}p_{z} & -3\sqrt{2}\gamma_{2}p_{+}p_{z} & -\sqrt{2}\gamma_{2}p_{\mathrm{eff}}^{2}\\ 0 & -\sqrt{3}\gamma_{2}p_{+}^{2} & 2\sqrt{3}\gamma_{2}p_{+}p_{z} & 2m_{0}\Delta_{\mathrm{SO}}+\gamma_{2}p_{\mathrm{eff}}^{2} & -\sqrt{6}\gamma_{2}p_{+}^{2} & \sqrt{6}\gamma_{2}p_{+}p_{z}\\ \sqrt{6}\gamma_{2}p_{+}p_{z} & \sqrt{2}\gamma_{2}p_{\mathrm{eff}}^{2} & -3\sqrt{2}\gamma_{2}p_{-}p_{z} & -\sqrt{6}\gamma_{2}p_{-}^{2} & 0 & 0\\ \sqrt{6}\gamma_{2}p_{+}^{2} & -3\sqrt{2}\gamma_{2}p_{+}p_{z} & -\sqrt{2}\gamma_{2}p_{\mathrm{eff}}^{2} & \sqrt{6}\gamma_{2}p_{-}p_{z} & 0 & 0 \end{array}\right)\;$$

where $p_{\mathrm{eff}}^{2}=\left(p_{x}^{2}+p_{y}^{2}-2p_{z}^{2}\right)$ and γ_1_ and γ_2_ are the Luttinger parameters.

The Hamiltonian ${\tilde{H}}_{1}$ is isotropic and while it affected the band dispersion it did not affect Bloch function mixing so it was dropped from discussion and the focus was on ${\tilde{H}}_{2}$. It was noted that the upper conduction bands were fourfold degenerate at *k* = 0 and have *J* = 3/2. These were labeled as heavy‐electrons with *J* = 3/2, *J*_*z*_ = ±3/2, and light‐electrons with *J* = 3/2, *J*_*z*_ = ±1/2. The lower spin‐orbit split off band has *J* = 1/2, *J*_*z*_ = ±1/2, and was the lowest conduction band.

The Luttinger Hamiltonian above is expressed in the basis of Bloch functions at the band‐edges, namely the kets |*J*, *J*_*z*_〉; in a bulk system the conduction band states will be described by Bloch waves of the form

(21) $$\begin{array}{c} \psi_{k}\left(r\right)=\frac{e^{i(k\cdot r)}}{\sqrt{V}}\sum_{J,J_{z}}C_{J,J_{z}}\left|J,J_{z}\right\rangle \end{array}$$

whose expansion coefficients were found by diagonalizing H(k) for a given wave vector.

The conduction bands in a perovskite slab are described by the Luttinger Hamiltonian above, but theinterest was specifically in the form of solutions appropriate for a slab geometry. Given a slab of area *A* assumed large, with thickness *d* centered at *z* = 0, the requirement that the wavefunction vanish at the slab surface constrained the eigenstates associated with the lowest slab subband in the slab to have the form

(22) $$\begin{array}{c} \psi_{1,k}\left(r\right)=\sqrt{\frac{2}{d}}\cos\left(\frac{\pi z}{d}\right)\frac{e^{i(k_{x}x+k_{y}y)}}{\sqrt{A}}\sum_{J,J_{z}}C_{J,J_{z}}\left|J,J_{z}\right\rangle \end{array}$$

where the $C_{J,J_{z}}$ again are expansion coefficients. This expression motivated a change of basis from the Bloch function basis,|*J*, *J*_*z*_〉, to what will be called the “slab” basis |*S*〉|*J*, *J*_*z*_〉, where $\left\langle z|S\right\rangle \equiv S\left(z\right)=\sqrt{\frac{2}{d}}\cos\left(\pi z/d\right)$. Transforming to this basis, the Hamiltonian ${\hat{H}}_{2}$ is represented by ${\tilde{H}}_{2}^{S}=\int_{d/2}^{d/2}d\mathrm{zS}\left(z\right){\tilde{H}}_{2}S\left(z\right)$ giving the following result

(23) $$\begin{array}{c} {\tilde{H}}_{2}^{S}=\frac{\hbar^{2}}{2m_{0}}\left(\begin{array}{cccccc} \gamma_{2}k_{\mathrm{tot}}^{2}+\frac{2m_{0}}{\hbar^{2}}\Delta_{\mathrm{SO}} & 0 & -\sqrt{3}\gamma_{2}k_{-}^{2} & 0 & 0 & \sqrt{6}\gamma_{2}k_{-}^{2}\\ 0 & \frac{2m_{0}}{\hbar^{2}}\Delta_{\mathrm{SO}}-\gamma_{2}k_{\mathrm{tot}}^{2} & 0 & -\sqrt{3}\gamma_{2}k_{-}^{2} & \sqrt{2}\gamma_{2}k_{\mathrm{tot}}^{2} & 0\\ -\sqrt{3}\gamma_{2}k_{+}^{2} & 0 & \frac{2m_{0}}{\hbar^{2}}\Delta_{\mathrm{SO}}-\gamma_{2}k_{\mathrm{tot}}^{2} & 0 & 0 & -\sqrt{2}\gamma_{2}k_{\mathrm{tot}}^{2}\\ 0 & -\sqrt{3}\gamma_{2}k_{+}^{2} & 0 & \gamma_{2}k_{\mathrm{tot}}^{2}+\frac{2m_{0}}{\hbar^{2}}\Delta_{\mathrm{SO}} & -\sqrt{6}\gamma_{2}k_{+}^{2} & 0\\ 0 & \sqrt{2}\gamma_{2}k_{\mathrm{tot}}^{2} & 0 & -\sqrt{6}\gamma_{2}k_{-}^{2} & 0 & 0\\ \sqrt{6}\gamma_{2}k_{+}^{2} & 0 & -\sqrt{2}\gamma_{2}k_{\mathrm{tot}}^{2} & 0 & 0 & 0 \end{array}\right) \end{array}$$

where $k_{\mathrm{tot}}^{2}=\left(-\frac{2\pi^{2}}{d^{2}}+k_{x}^{2}+k_{y}^{2}\right)$. All linear order terms involving *p*_*z*_ vanished. This slab Hamiltonian can be broken into two pieces as follows

(24) $$\begin{array}{c} {\tilde{H}}_{2}^{S}={\tilde{H}}_{2}^{S}\left(0\right)+{\tilde{H}}_{2}^{S}\left(k\right)\; \end{array}$$

where,

(25) $$\begin{array}{c} {\tilde{H}}_{2}^{S}\left(k\right)=\frac{\hbar^{2}}{2m_{0}}\left(\begin{array}{cccccc} \gamma_{2}k_{⊥}^{2} & 0 & -\sqrt{3}\gamma_{2}k_{-}^{2} & 0 & 0 & \sqrt{6}\gamma_{2}k_{-}^{2}\\ 0 & -\gamma_{2}k_{⊥}^{2} & 0 & -\sqrt{3}\gamma_{2}k_{-}^{2} & \sqrt{2}\gamma_{2}k_{⊥}^{2} & 0\\ -\sqrt{3}\gamma_{2}k_{+}^{2} & 0 & -\gamma_{2}k_{⊥}^{2} & 0 & 0 & -\sqrt{2}\gamma_{2}k_{⊥}^{2}\\ 0 & -\sqrt{3}\gamma_{2}k_{+}^{2} & 0 & \gamma_{2}k_{⊥}^{2} & -\sqrt{6}\gamma_{2}k_{+}^{2} & 0\\ 0 & \sqrt{2}\gamma_{2}k_{⊥}^{2} & 0 & -\sqrt{6}\gamma_{2}k_{-}^{2} & 0 & 0\\ \sqrt{6}\gamma_{2}k_{+}^{2} & 0 & -\sqrt{2}\gamma_{2}k_{⊥}^{2} & 0 & 0 & 0 \end{array}\right)\; \end{array}$$

Here, $k_{⊥}^{2}=\left(k_{x}^{2}+k_{y}^{2}\right)$. The more interesting part is ${\tilde{H}}_{2}^{S}\left(0\right)$

(26) $$\begin{array}{c} {\tilde{H}}_{2}^{S}\left(0\right)=\left(\begin{array}{cccccc} \frac{\delta }{3}+\Delta_{SO} & 0 & 0 & 0 & 0 & 0\\ 0 & \Delta_{SO}-\frac{\delta }{3} & 0 & 0 & \frac{\sqrt{2}\delta }{3} & 0\\ 0 & 0 & \Delta_{SO}-\frac{\delta }{3} & 0 & 0 & -\frac{\sqrt{2}\delta }{3}\\ 0 & 0 & 0 & \frac{\delta }{3}+\Delta_{SO} & 0 & 0\\ 0 & \frac{\sqrt{2}\delta }{3} & 0 & 0 & 0 & 0\\ 0 & 0 & -\frac{\sqrt{2}\delta }{3} & 0 & 0 & 0 \end{array}\right) \end{array}$$

where the parameter δ can be considered as an effective crystal field, and is given by,

(27) $$\begin{array}{c} \delta =-3\frac{\hbar^{2}}{2m_{0}}\gamma_{2}\frac{2\pi^{2}}{d^{2}}\; \end{array}$$

## Conflict of Interest

The authors declare no conflict of interest.

## Supporting information

Supporting Information

## Data Availability

Research data are not shared.
