# Supplementary material for: Dark and Bright Excitons in Halide Perovskite Nanoplatelets
Source: Adv Sci (Weinh). 2021 Dec 23;9(5):2103013. doi: 10.1002/advs.202103013 (PMC8844578; doi:10.1002/advs.202103013)
Supplement: Supplementary file 1 — Supporting Information [file ADVS-9-2103013-s001.pdf]

ADVANCED SCIENCE
Open Access

Supporting Information

for *Adv. Sci.,* DOI: 10.1002/advs.202103013

Dark and Bright Excitons in Halide Perovskite Nanoplatelets

Moritz Gramlich, Michael W. Swift, Carola Lampe, John L. Lyons, Markus Döblinger, Alexander L. Efros, Peter C. Sercel,* and Alexander S. Urban**

Supporting Information:
Dark and Bright Excitons in Halide Perovskite Nanoplatelets

Moritz Gramlich[a],[1] Michael W. Swift[a],[2] Carola Lampe,[1] John L. Lyons,[2] Markus Döblinger,[3] Alexander L. Efros,[2] Peter C. Sercel,[4,5] and Alexander S. Urban[1]

[1]*Nanospectroscopy Group, Nano-Institute Munich, Department of Physics, Ludwig-Maximilians-Universität, Munich 80539, Germany*

[2]*Center for Computational Materials Science, U.S. Naval Research Laboratory, Washington D.C. 20375, United States*

[3]*Department of Chemistry, Ludwig-Maximilians-Universität (LMU) & Center for NanoScience (CeNS), 81377 Munich, Germany*

[4]*Department of Applied Physics and Materials Science, California Institute of Technology, Pasadena, California 91125, United States*

[5]*Center for Hybrid Organic Inorganic Semiconductors for Energy, Golden, Colorado 80401, United States*

CONTENTS

List of Figures 2

Supporting Methods 16

1. Variational determination of internal wavefunction 16
2. Slab model of confinement 18
3. Short-range exchange 19
4. Long-range exchange 20
5. Three-level decay model 22
6. Hybrid DFT calculations of nanoplatelet electronic structure 24

Supporting References 26

[a] Contributed equally

LIST OF FIGURES

1 STEM-HAADF images of caesium lead bromide nanoplatelets (NPLs) drop-casted onto TEM grids . . . 3
2 Optical properties of NPL dispersions in hexane . . . 4
3 Temperature-resolved PL spectra . . . 5
4 Reproducibility of PL measurements . . . 6
5 Gauss-fitting of Jump . . . 6
6 Two-level model fit . . . 7
7 Variational determination of internal wavefunction . . . 8
8 Thickness-dependent gap and transition energy . . . 9
9 Dimensionless long-range exchange integrals . . . 10
10 PL spectra around jump . . . 10
11 PL decay curves . . . 11
12 Temperature resolved PL spectra of the 2ML NPLs . . . 12
13 Theoretical temperature-resolved PL spectra . . . 13
14 First-principles results . . . 15

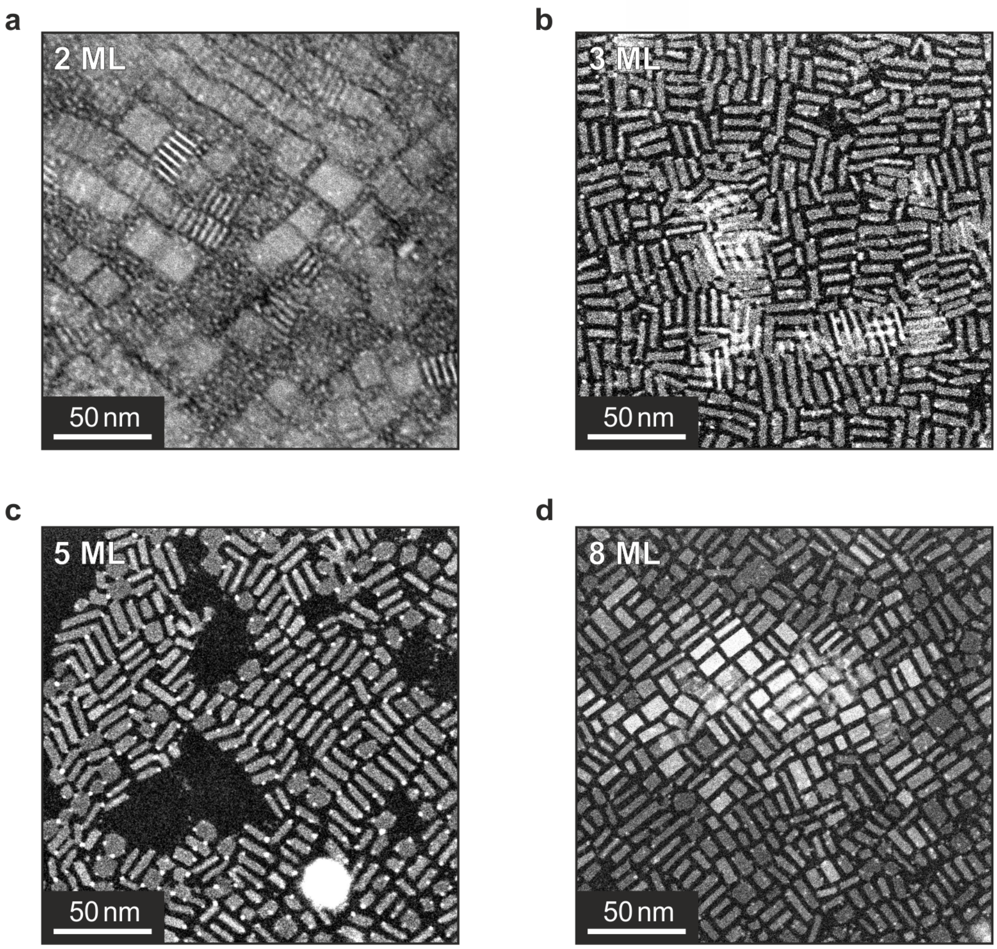

Supporting Figure 1. **STEM-HAADF images of caesium lead bromide nanoplatelets (NPLs) drop-casted onto TEM grids.** (**a**) 2 monolayer (ML), (**b**) 3ML, (**c**) 5ML and (**d**) 8ML NPLs.

Sample	d (nm)	$\sigma(d)$ (nm)	L_x (nm)	$\sigma(L_x)$ (nm)	L_y (nm)	$\sigma(L_y)$(nm)
2ML	1.5	0.2	14.0	1.6	16.4	2.7
3ML	2.5	0.5	15.1	4.3		
5ML	3.3	0.6	14.9	2.4		
8ML	4.5	1.1	12.6	3.5		

Supporting Table 1. Dimensions d, L_x, and L_y of NPLs and their standard deviations σ, deduced from STEM-HAADF images.

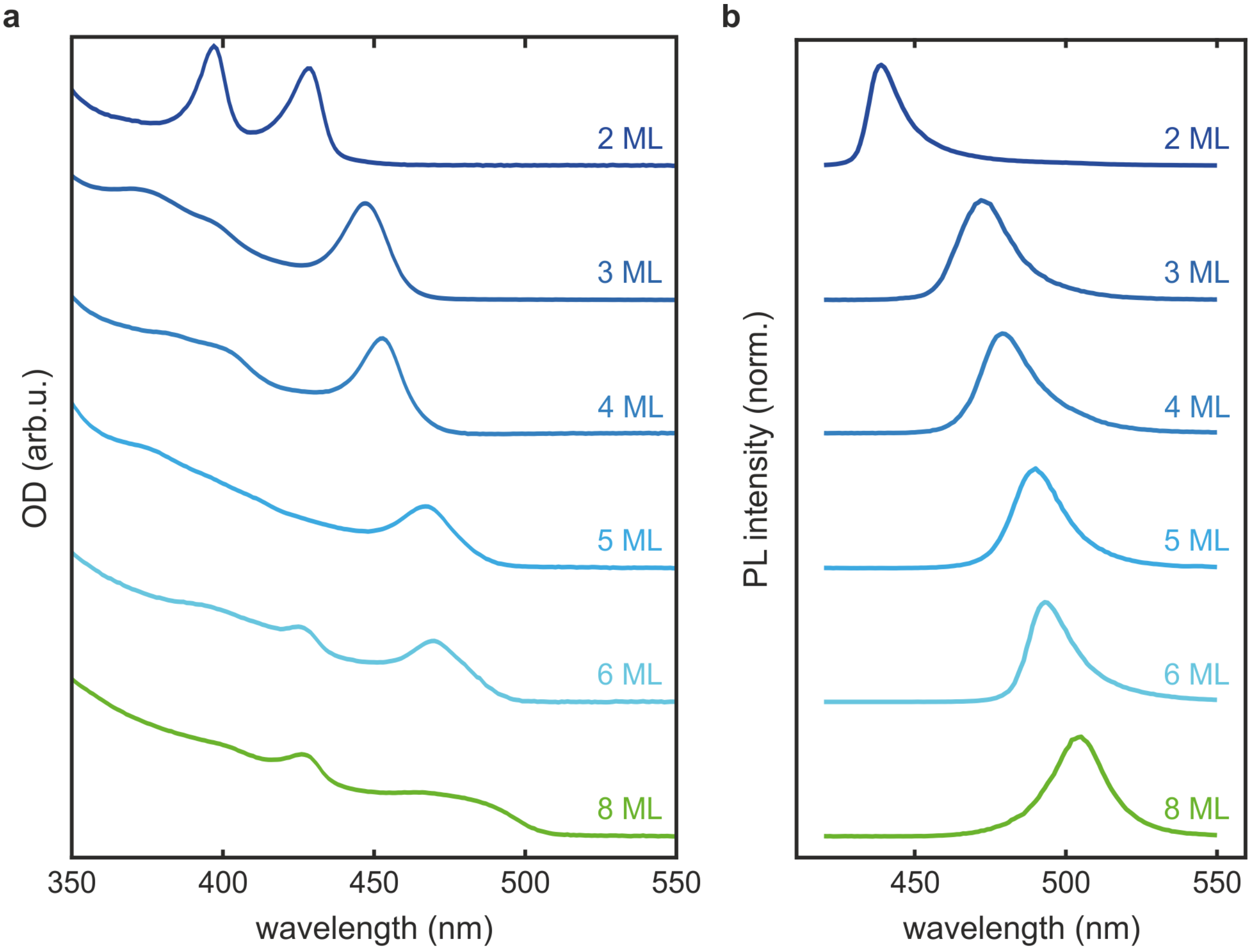

Supporting Figure 2. **Optical properties of NPL dispersions in hexane.** (**a**) Absorption and (**b**) PL spectra. The spectra are offset vertically for clarity. PL spectra were normalized to the emission maximum.

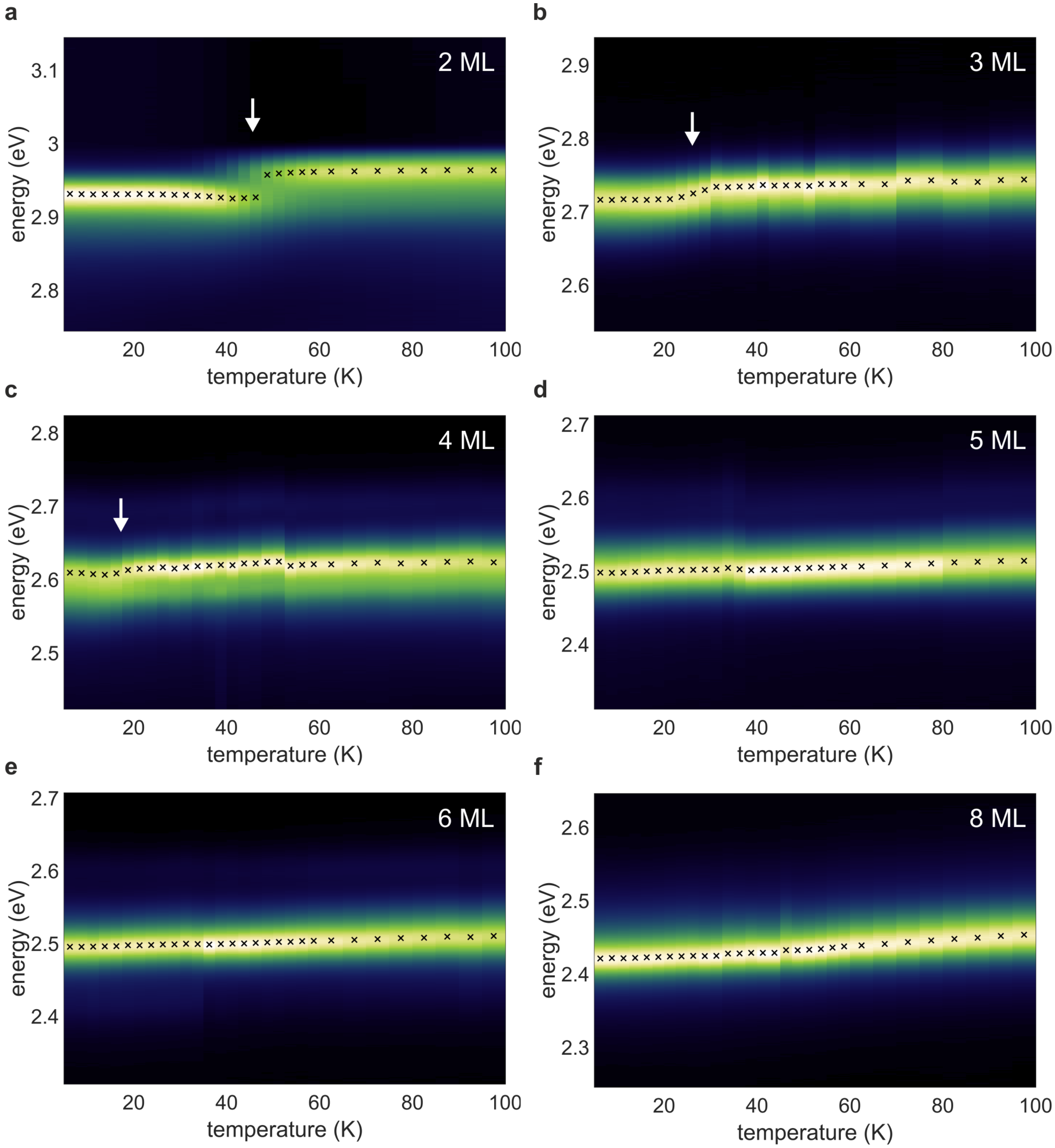

Supporting Figure 3. **Temperature-resolved PL spectra.** Thin-films of caesium lead bromide NPLs were drop-cast onto SiO_2-coated silicon substrates, and PL spectra acquired between 4 K and 100 K. The individual spectra are normalized to the overal integrated intensity. For clarity, the positions of the respective maxima for each temperature are denoted by black crosses. The PL jumps for the 2ML, 3ML, and 4ML samples are marked by white arrows.

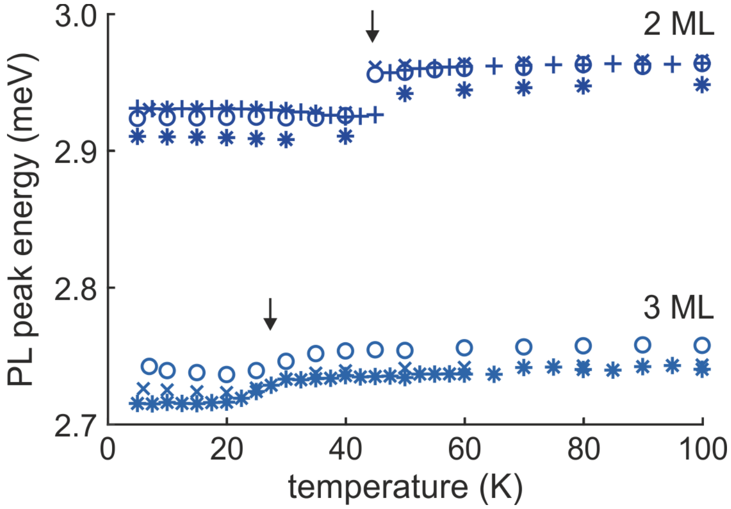

Supporting Figure 4. **Reproducibility of PL measurements.** To verify the temperature at which the jump in the PL takes place, we synthesized four batches of 2ML and three batches of 3ML NPLs, prepared thin films and acquired their temperature-dependent PL spectra between 4 K and 100 K. While there are slight variations in the overall PL positions from batch to batch, the temperature at which the jump takes place is extremely reproducible for both NPL thicknesses (marked by black arrows). The dark symbols denote the 2ML samples and the lighter blue symbols the 3ML samples. Within each color, the different symbols denote different batches.

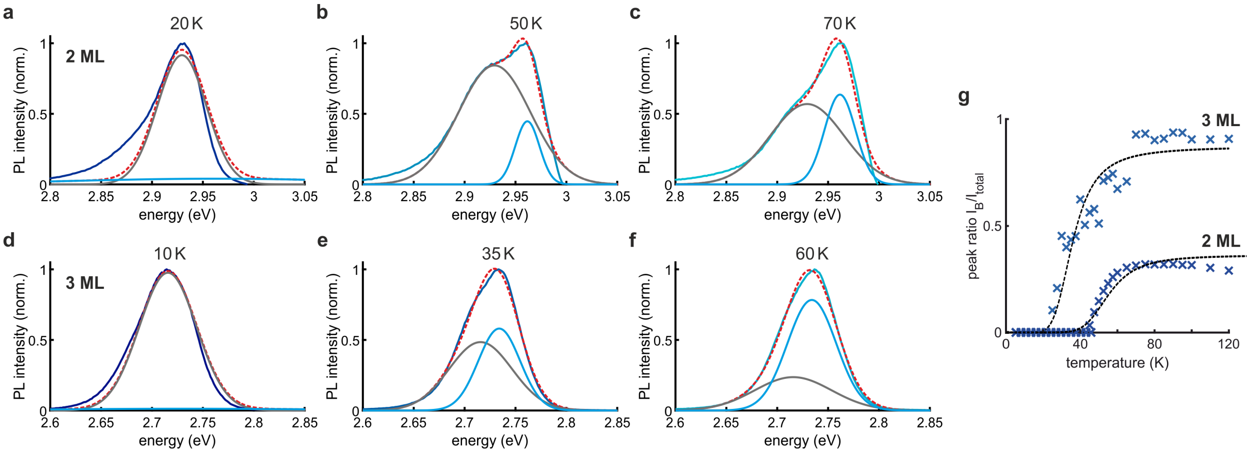

Supporting Figure 5. **Double Gaussian fits of 2ML and 3ML spectra.** a) - f) The spectra around the jump can nicely be reproduced by a sum of two Gaussians fixed to the energetic positions corresponding to the dark and a bright level. g) The temperature-induced shift of the ratio between the two Gaussians displays Boltzmann activation behaviour.

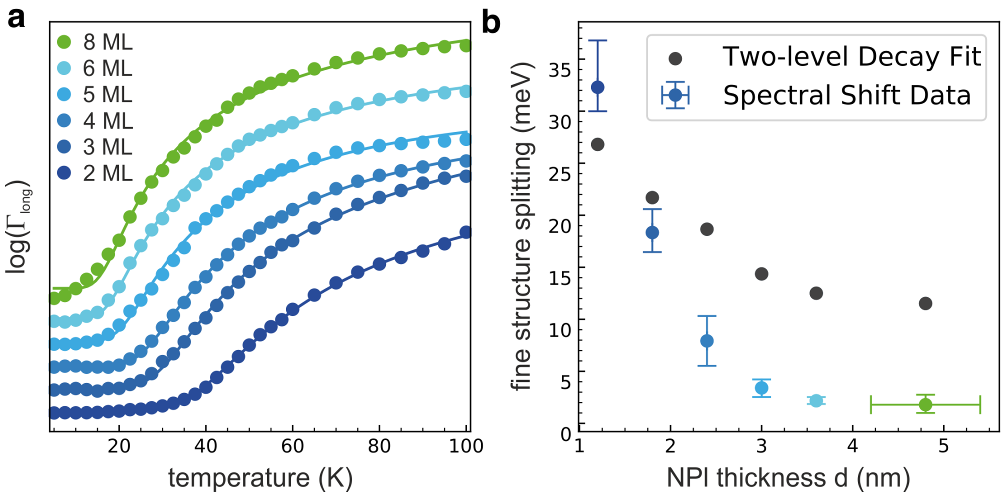

Supporting Figure 6. **Fit of the slow PL decay component accordingto the two-level kinetic model.** (**a**) Two-level four-parameter fit (see Ref. [1]) to the PL decay data for 2ML - 8ML NPLs. (**b**) Fitted energies from the two-level model (black points) compared to the spectral shift data (colored points). The two-level model gives incorrect bright level energies. This illustrates the need to expand to a three-level model that takes both bright levels (in-plane and out-of-plane) as well as the dark level into account.

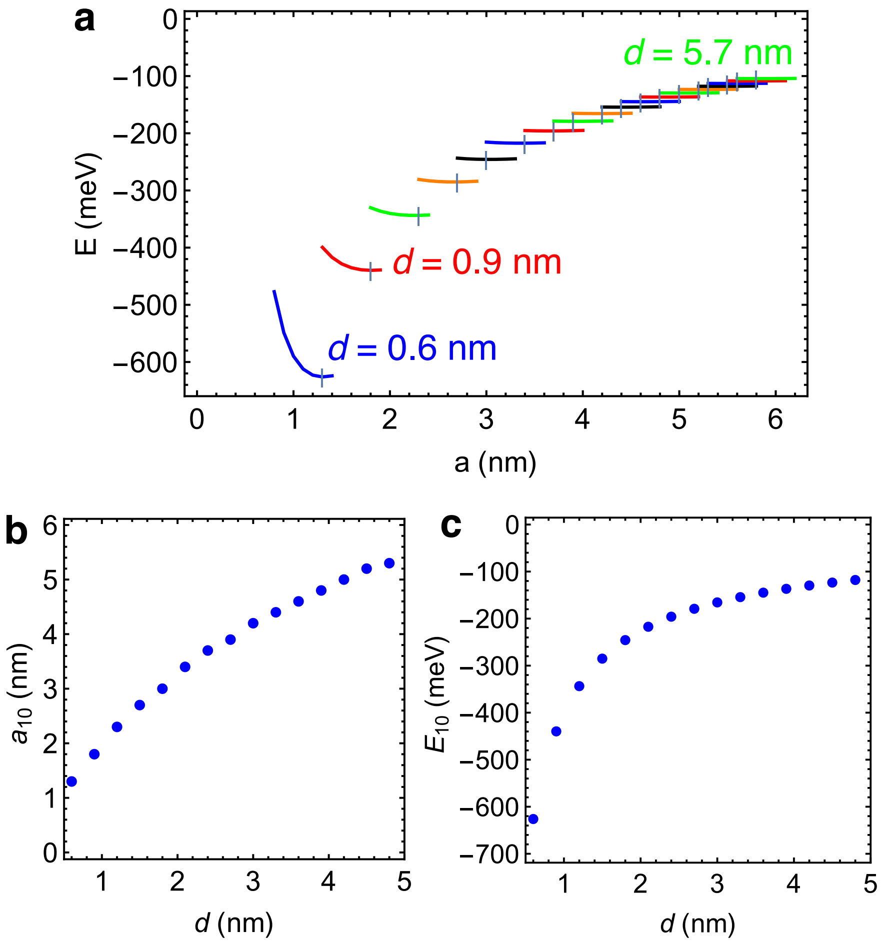

Supporting Figure 7. **Variational determination of internal wavefunction.** (**a**) Energy of the 1s variational wavefunction Eq. (2) in the interaction potential Eq. (S2) as a function of variational parameter a, shown for varying layer thickness d from 0.6 nm to 5.7 nm in steps of 0.3 nm. The minima at $a = a^*$ are indicated for each curve. (**b**) The resulting effective radius versus NPL thickness d. (**c**) Exciton 1s binding energy versus NPL thickness d.

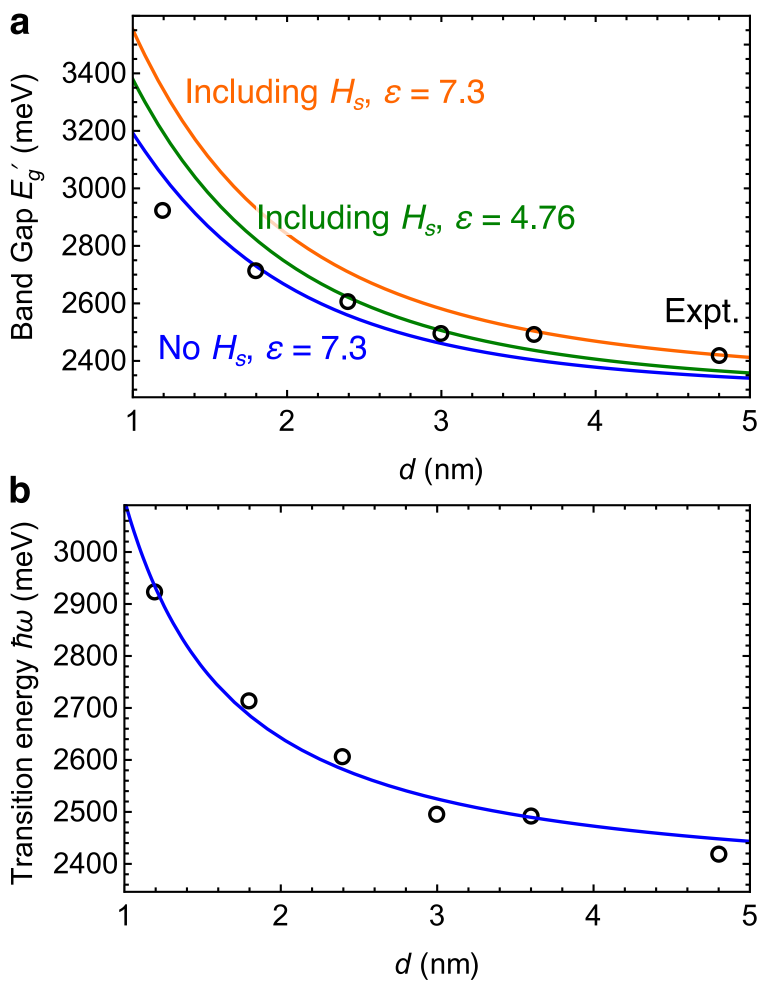

Supporting Figure 8. **Thickness-dependent gap and transition energy.** (**a**) Thickness-dependent gap E_g' vs thickness d (see Supporting Methods 2), shown without self-interaction H_s (Eq. (S9)) in blue, with H_s in orange, and with H_s using the high-frequency dielectric constant $\varepsilon = 4.76$ in green. (**b**) Empirical fit for the transition energy $\hbar\omega$ (blue line) to the PL peak positions (black circles). The physically motivated functional form is $E = A + C/d^2 + \left[B/(D + 1/d^2)\right]/d$, with fitted values $A = 2241$, $B = 2685$, $C = 165.9$, and $D = 3.269$. The constant term corresponds to a bulk gap, $1/d^2$ corresponds to a kinetic confinement term, and $1/d$ corresponds to a Coulomb term. Note that the d-dependence in the $1/d$ Coulomb-like term implies a d-dependent dielectric function.

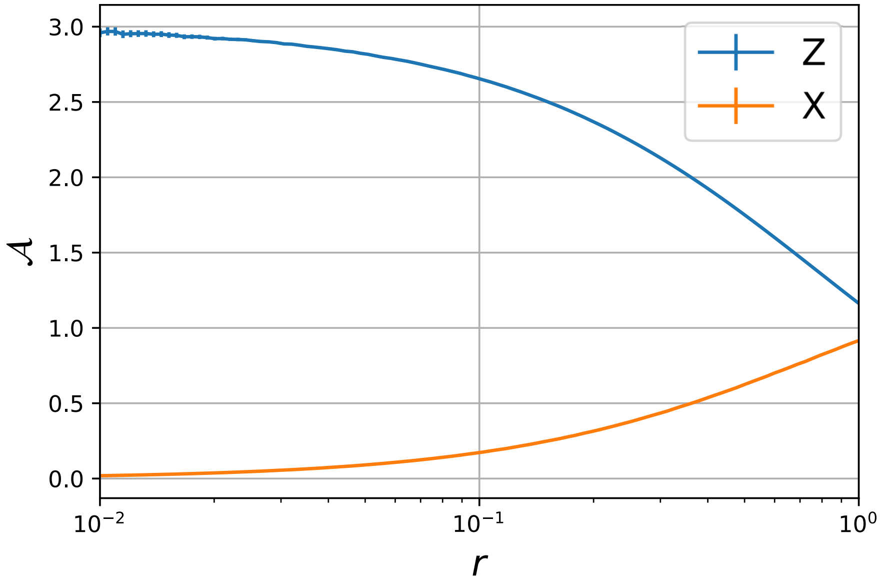

Supporting Figure 9. **Dimensionless long-range exchange integrals.** $\mathcal{A}_Z$ and $\mathcal{A}_X$ in a square nanoplatelet as a function of aspect ratio $r = d/L$. These numerical results recover the known limiting case: $\mathcal{A}_Z \to 3$ and $\mathcal{A}_X \to 0$ as $r \to 0$. Statistical errors from the stochastic integration method are shown by error bars corresponding to five standard deviations. Error bars are smaller than the line width for most r values.

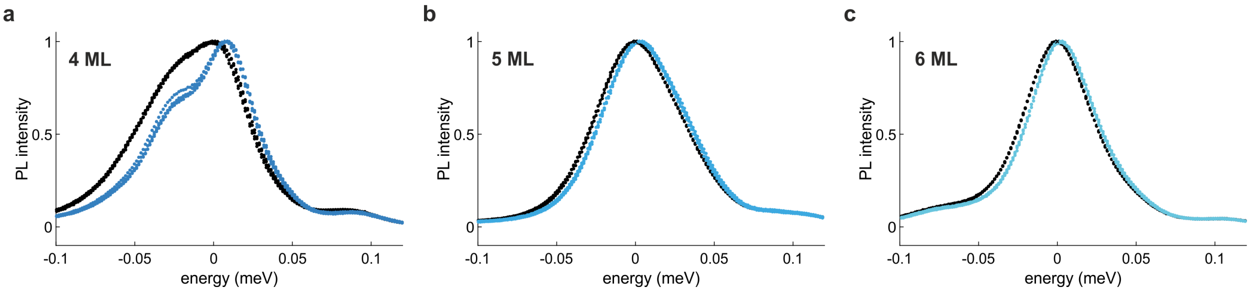

Supporting Figure 10. **PL spectra of 4ML, 5ML and 6ML NPLs.** The PL spectra shown in Figure 3b of the main text enlarged for the 4ML, 5ML and 6ML NPLs. For the temperature intervals displayed refer to Supporting Table 2.

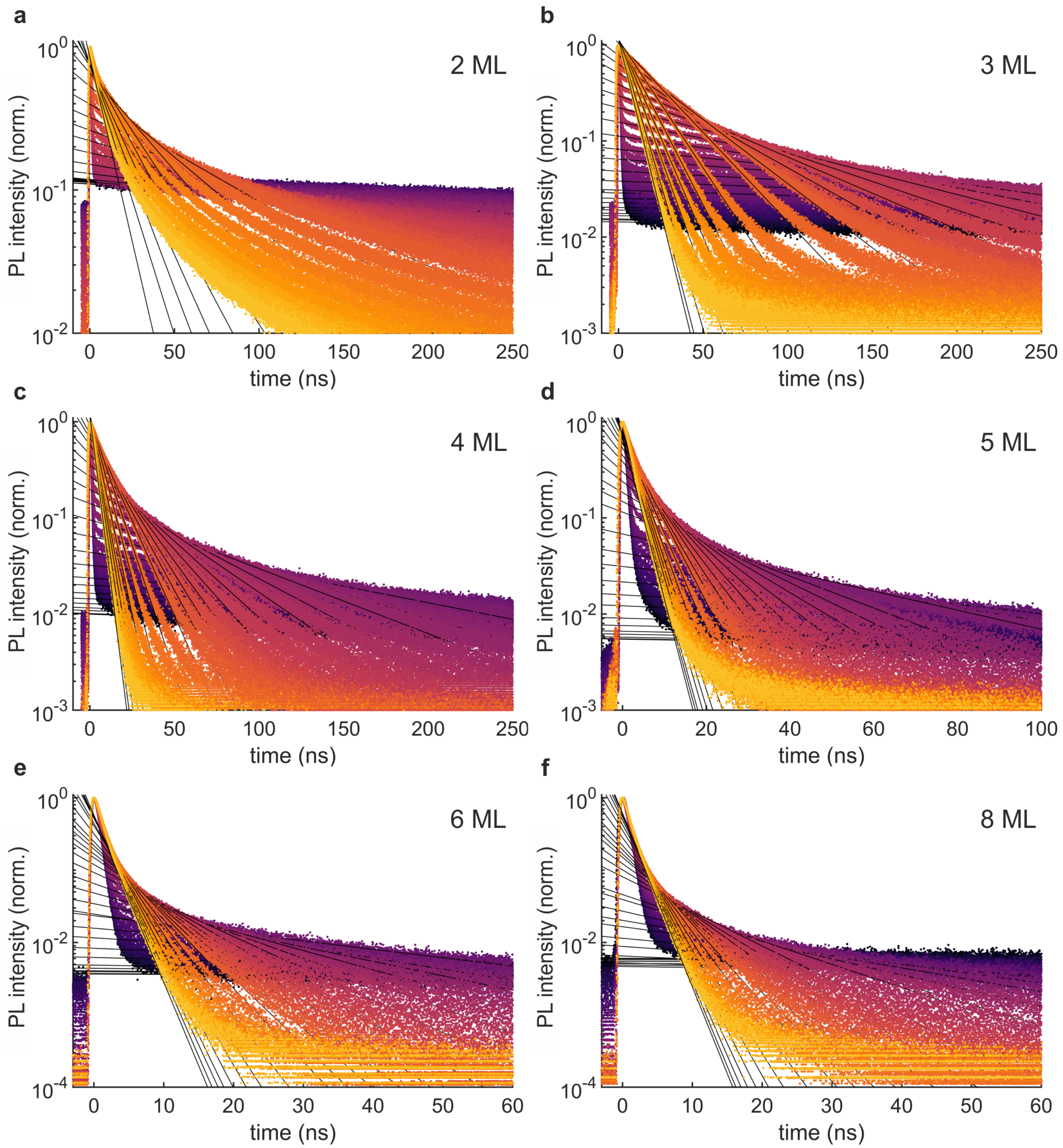

Supporting Figure 11. **Temperature-dependence of the PL decay of caesium lead bromide NPLs**. For each thickness, the decay curves are normalized to the maximum and color coded according to temperature from black at 4 K to yellow at 100 K. The black line indicates the exponential fitting of the slower decay component to extract the corresponding rate Γ_{long}.

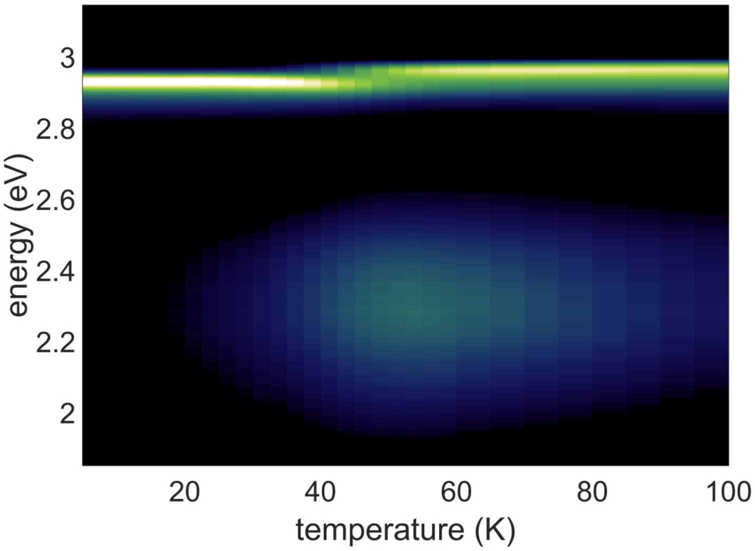

Supporting Figure 12. **Temperature-resolved PL spectra of the 2ML NPLs.** Extended over a larger spectral range (1.8-3.2 eV), a clear, broad contribution from a self-trapped exciton centered at 2.3 eV and between 30 K and 90 K can be seen. Exciton transfer from the dark and bright state to the self-trapped exciton will modify the decay times observed.

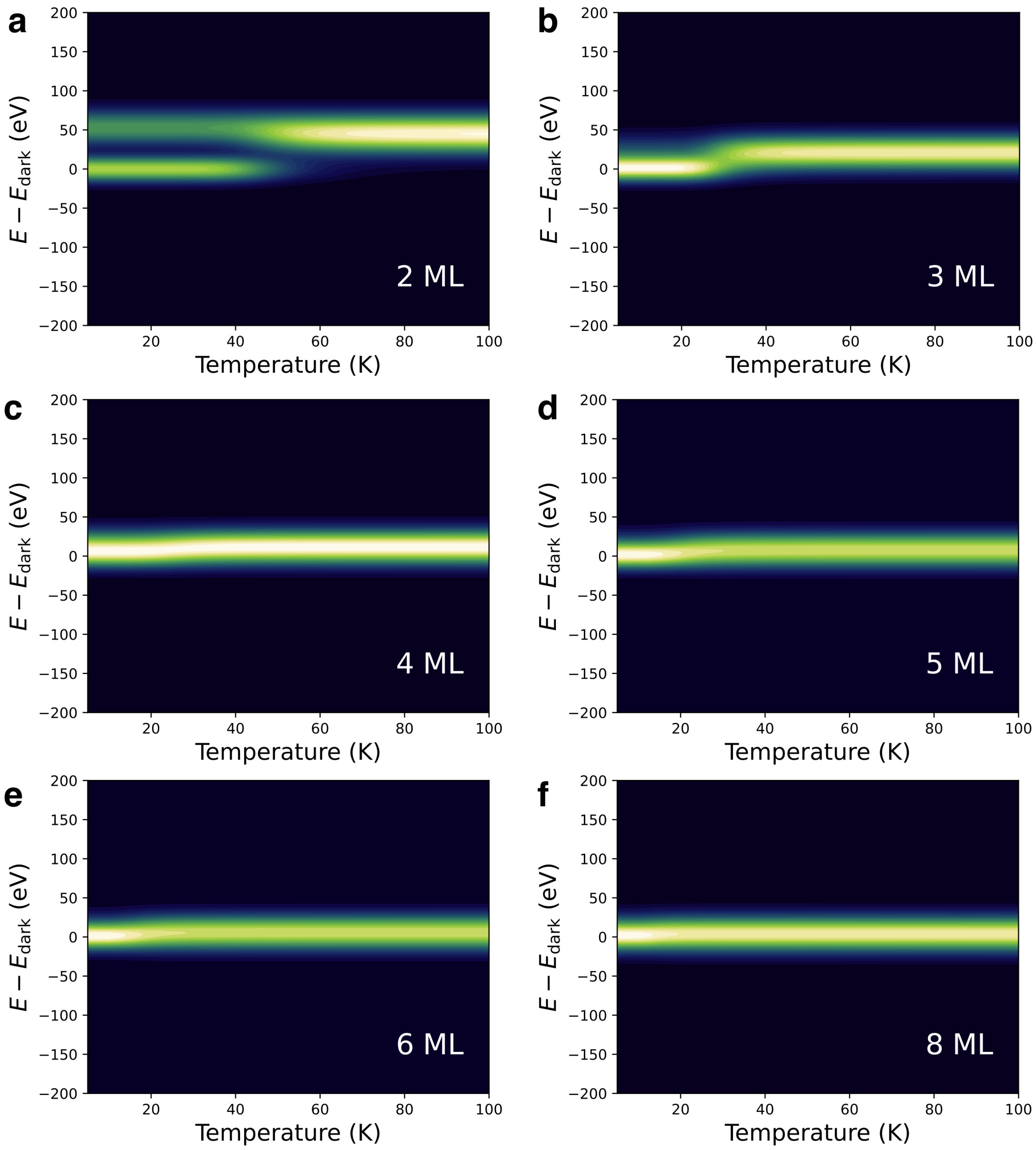

Supporting Figure 13. **Theoretical temperature-resolved PL spectra,** shown for comparison to Supporting Fig. 3. Spectra are calculated based on the fitted rates in Supporting Table 3 using Eq. S35, assuming a full-width at half-maximum of 35 meV for the dark state and 25 meV for the bright states. Just as in the experimental data, there is a clear "jump" in the 2ML, 3ML, and 4ML samples as temperature increases, corresponding to the increased population of the bright states. Note that in panel a, the bright state still contributes to the PL at low temperature due to a fitting artifact: the fit adjusts for the overestimation of ΔE_2 by decoupling that state from the other two (see γ_{2B} and γ_{21} in Supporting Table 3). If wavefunction leakage were taken into account, ΔE_2 would be smaller, γ_{2B} and γ_{21} would be larger, and the bright state PL would vanish at low temperatures as in the other panels.

NPL thickness	2 ML	3 ML	4 ML	5 ML	6 ML	8 ML
Temp. before PL shift	37.5 K	17.5 K	12.5 K	10 K	10 K	10 K
Temp. after PL shift	57.5 K	30 K	22.5 K	17.5 K	17.5 K	17.5 K
ΔE_{BGR}	3.9 meV	2.6 meV	2.0 meV	1.7 meV	–	–

Supporting Table 2. **Temperature intervals before and after PL jump.** The temperatures of the last spectrum before the PL jump and the first one after the jump are given. Several spectra before and after these intervals are used to calculate the bright-dark splitting. The interval shifts to lower temperatures with increasing thickness and becomes smaller. The corresponding shift of the PL emission over this interval resulting from band gap renormalization, ΔE_{BGR} .[2]

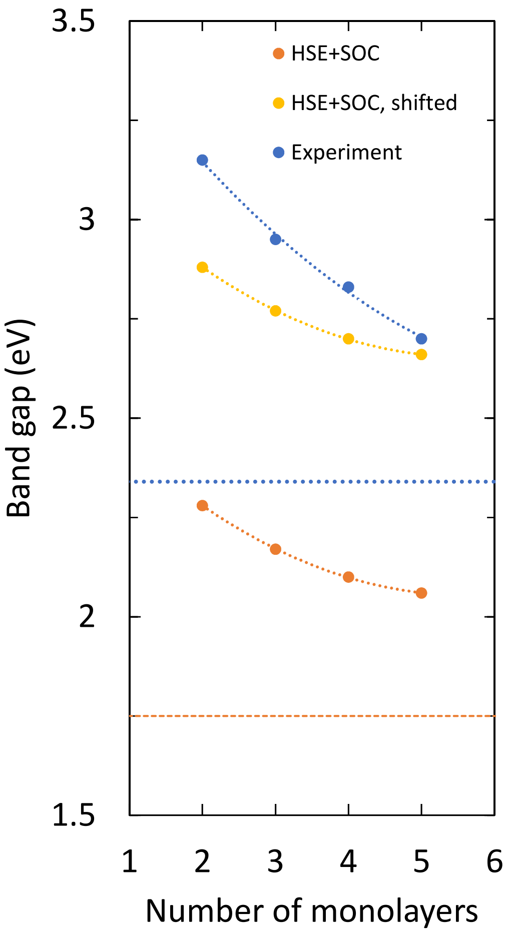

Supporting Figure 14. **First-principles results.** DFT-calculated band gaps of $CsPbBr_3$ nanoplatelets as a function of thickness, in comparison with experiment. Yellow data are shifted upwards by 0.6 eV, equal to the difference between the calculated bulk band gap and the value used in the effective mass modeling.

Sample	Γ_D	Γ_{B1}	γ_{1D}	γ_{2D}	γ_{21}	Γ_{B2}	ΔE_1	ΔE_2
2 ML	0.001195	9.2023	7220.27	3.460e−19	7.59895e−14	377.499	37.1802	52.8658
3 ML	0.0006465	1.7621	1.36439	118.396	4.03375	0.100598	20.982	32.1628
4 ML	0.001444	1.31547	0.0756467	15.876	75.3483	0.116409	11.5309	24.683
5 ML	0.001852	1.15747	0.0356909	2979.06	4.93414	0.131462	7.59075	18.6558
6 ML	0.001267	1.94283	0.0147374	105.374	1.82447	0.257157	5.57448	14.3015
8 ML	0.0007738	190.21	2.804e−34	0.885462	30.1677	29.7665	3.58084	8.83464

Supporting Table 3. **Fitted decay parameters.** Parameters of the decay model Γ_D , Γ_{B1} , γ_{1D} , γ_{2D} , and γ_{21} fit to the experimental data. Also shown are ΔE_1 and ΔE_2 , which were not fit but rather fixed to the theoretical values, and Γ_{B2} which is fixed based on the fitted Γ_{B1} and the calculated ratio of radiative decay rates. All parameters are in ns^{-1} except ΔE_1 and ΔE_2 , which are in meV. Note that the high radiative rates fitted to the 2ML and 8ML data are fitting artifacts. In the 2ML case this arises from the overestimation of the exchange interaction connected with the hard-wall boundary conditions. In the 8ML case, it arises from the additional contribution of decay from 7ML and 9ML NPLs which coexist in the nominally 8ML sample.

SUPPORTING METHODS

1. Variational determination of internal wavefunction

Dielectric confinement significantly affects the wavefunction of the exciton internal motion. Ritova and Keldysh derived the analytical form of the Coulomb potential created by a point charge located in a dielectric slab. The slab is embedded in a medium of dissimilar dielectric constant, which depends strongly on the slab thickness, d. If the electron-hole separation in the slab plane $\boldsymbol{\rho} = \boldsymbol{\rho}_e - \boldsymbol{\rho}_h$ can be considered to be much larger then the slab thickness, d, this potential is significantly simplified and can be written as: [3, 4]

$$V_d^{2d}(\boldsymbol{\rho}) = \frac{\pi e}{\varepsilon_i d}\left(\mathbf{H}_0(\rho/r_0) - Y_0(\rho/r_0)\right) , \tag{S1}$$

where $\mathbf{H}_0$ is the zeroth-order Struve function, Y_0 is the zeroth-order Bessel function of the second kind (a.k.a. the Neumann function), ε_i and ε_o are the interior and exterior dielectric constants respectively, whose ratio $\kappa = \varepsilon_i/\varepsilon_o$ further determines $r_0 = d\kappa/2$.

In nanoplatelets (NPLs) of several ML thickness, for which the inorganic layer thickness is comparable to or larger than the average electron-hole separation (characterized by an effective exciton radius on the order of $1\ \text{nm}$ to $3\ \text{nm}$) the approximate Eq. (S1) is no longer valid. In this case, for calculations of the exciton binding energy and the wavefunction it is more convenient to use a potential form derived using the image charge method by Hanamura *et al.* [5] This approach was previously utilized to calculate exciton binding energies in layered 2D hybrid organic-inorganic perovskites by Hong *et al.*[6] In terms of the relative radial coordinate of the electron and hole in the plane, ρ, and the z-coordinates $z_{e(h)}$ of the electron and hole, the interaction potential is

$$V_{eh}^{3d}(\rho, z_e, z_h) = -\frac{e^2}{\varepsilon_i}\sum_{n=-\infty}^{n=\infty}\frac{q_n}{\sqrt{\rho^2+(z_e-z_{h,n})^2}}\ , \tag{S2}$$

where $q_n = q_{-n} = [(\kappa-1)/(\kappa+1)]^{|n|}$ and $z_{h,n} = (-1)^{|n|}z_h + nd$.

Since the full interaction potential in Eq. (S2) is fully three-dimensional, we will use the adiabatic separation of variables which assumes that the motion perpendicular to the NPL layer is much faster than the relative electron-hole motion in NPL plane. Within this assumption we can derive the adiabatic Coulomb potential $V_d(\rho)$ describing the relative electron-hole motion by averaging Eq. (S2) over the wavefunctions of electrons $\psi_c(z_e) = \sqrt{2/d}\cos(\pi z_e/d)$ and holes $\psi_v(z_h) = \sqrt{2/d}\cos(\pi z_h/d)$ confined in NPLs, respectively:

$$V_d^{Ad}(\rho) = \int_{-d/2}^{d/2}\int_{-d/2}^{d/2} dz_e dz_h \psi_c^*(z_e)\psi_v^*(z_h)V_{eh}^{3d}(\rho, z_e, z_h)\psi_c(z_e)\psi_v(z_h)\ . \tag{S3}$$

The effective Hamiltonian describing the electron-hole relative motion in NPLs is given by

$$\hat{H}_{\text{REL}} = -\frac{\hbar^2\nabla_\rho^2}{2\mu} + V_d^{Ad}(\rho)\ . \tag{S4}$$

There are no analytical solutions for the adiabatic Hamiltonian $\hat{H}_{\text{REL}}$. To find the binding energy and the wavefunction of the ground state we use the variational procedure with an ansatz function described in Eqs. (2), (3). This allows us to find the dependence of the effective radius of 2D exciton and its binding energy as a function of NPL thickness.

The variation procedure using the 2D Ritova-Keldysh potential from Eq. (S1) gives a qualitatively correct behavior, but $|\phi_d(0)|^2$, and thus the exchange energies, are significantly underestimated.

2. Slab model of confinement

The NPL band gap E_g' varies with thickness due to quantum confinement. We find the energy as a function of NPL thickness by solving an 8-band K.P Hamiltonian taking wave vector $k_z = \pi/d$ in the direction perpendicular to the plane of the NPL. The 8-band K.P Hamiltonian decomposes into two degenerate sub-blocks as follows:

$$\tilde{H}^{\pm}(k_z) = \begin{pmatrix} \tilde{H}^{+}(k_z) & 0 \\ 0 & \tilde{H}^{-}(k_z) \end{pmatrix} , \tag{S5}$$

where the degenerate sub-blocks are given, with the corresponding Bloch function basis for each sub-block, by:

$$\begin{matrix} & |3/2,\pm 3/2\rangle & |3/2,\mp 1/2\rangle & |1/2,\mp 1/2\rangle & |1/2,\mp/2\rangle \end{matrix}$$

$$\tilde{H}^{\pm}(k_z) = \begin{pmatrix} \Delta_{SO} + E_g + \frac{\left(\gamma_1^0 - 2\gamma_2^0\right)\hbar^2 k_z^2}{2m_0} & 0 & 0 & 0 \\ 0 & \Delta_{SO} + E_g + \frac{\left(\gamma_1^0 + 2\gamma_2^0\right)\hbar^2 k_z^2}{2m_0} & \pm\sqrt{2}\gamma_2^0 \frac{\hbar^2 k_z^2}{m_0} & -iP_{cv}\frac{\sqrt{2}\hbar k_z}{\sqrt{3}m_0} \\ 0 & \pm\sqrt{2}\gamma_2^0 \frac{\hbar^2 k_z^2}{m_0} & E_g + \gamma_1^0 \frac{\hbar^2 k_z^2}{2m_0} & \mp iP_{cv}\frac{\hbar k_z}{\sqrt{3}m_0} \\ 0 & iP_{cv}\frac{\sqrt{2}\hbar k_z}{\sqrt{3}m_0} & \pm iP_{cv}\frac{\hbar k_z}{\sqrt{3}m_0} & -Q\frac{\hbar^2 k_z^2}{2m_0} \end{pmatrix} , \tag{S6}$$

Eq. (S5) is diagonalized as a function of $k_z = \pi/d$, where d is the thickness of the NPL, using parameters $Eg = 2.342$ eV, $E_p = 27.88$ eV, $\Delta_{SO} = 1.5$ eV from Table 1 and parameters $Q = -5.36$, $\gamma_1^0 = 0.5$, and $\gamma_2^0 = 0$, reflecting remote band contributions to the effective mass. These parameters are chosen to reflect the low temperature band gap and the band-edge reduced effective mass of the exciton measured in bulk $CsPbBr_3$ thin films by Yang *et al.*[7] From this procedure we obtain the NPL band gap, $E_g'(d)$. It can be shown based on 8-band $k \cdot p$ theory [8] that the electron and hole masses can be written,

$$\frac{1}{m_h} = \frac{1}{3}\left(\frac{2E_p}{\Delta_{SO} + E_g} + \frac{E_p}{E_g}\right) + Q \qquad \frac{1}{m_e} = \frac{E_p}{3E_g} + \gamma_1^0 . \tag{S7}$$

Using the nanoplatelet thickness dependent band gap $E_g'(d)$ in place of E_g in these expressions, we find the dependence of the reduced effective mass on the NPL thickness:

$$\frac{1}{\mu(d)} = Q + \gamma_1^0 + \frac{1}{3}\left(\frac{2E_p}{\Delta_{SO} + E_g'(d)} + \frac{E_p}{E_g'(d)}\right) + \frac{E_p}{3E_g'(d)} , \tag{S8}$$

The slab-thickness dependent gap $E_g'(d)$ value as defined above is then also used to correct the Luttinger parameter γ_2 using $\gamma_2 = \gamma_2^0 + E_p/6E_g'(d)$ and assuming $\gamma_2^0 = 0$ (see Eq. (5)).

To obtain the PL transition energy $\hbar\omega$, the binding energy and self-energy of the electron and hole must also be included. The self-energy may be approximated by

$$H_s = \frac{e^2}{2\varepsilon} \sum_{n\neq 0} q_n \left(\frac{1}{|z_e - z_{e,n}|} + \frac{1}{|z_h - z_{h,n}|} \right) , \tag{S9}$$

where q_n and z_n are defined as in Eq. (S2). This prediction is compared with the experimental PL peaks in Supporting Fig. 8a. Comparison of the measured PL peak energies with the model curves suggests that the effective dielectric constant increases with decreasing slab thickness, approaching the high-frequency dielectric constant in thin NPLs. Further discrepancies are likely due to the failure of hard-wall boundary conditions due to wavefunction leakage in the thinnest NPLs. Since these effects are beyond the scope of this work, we use a physically motivated empirical fit for $\hbar\omega$: see Supporting Fig. 8b.

3. Short-range exchange

We now calculate the effect of the Bloch function anisotropy on the short-range exchange. In the pair electron-hole basis representation the exciton Bloch functions can be expressed as $|P\rangle \equiv (u_{c,1/2}u_{v,1/2}, u_{c,1/2}u_{v,-1/2}, u_{c,-1/2}u_{v,1/2}, u_{c,-1/2}u_{v,-1/2})$ As discussed above, it is more convenient to transform $|P\rangle$ into a basis where the transition dipoles are aligned along the X, Y, Z directions. We call this the $\mathcal{O}$ basis, and denote the Bloch pairs in this basis as $|X_i\rangle$ running over $|D\rangle$, $|X\rangle$, $|Y\rangle$, $|Z\rangle$, which are the dark state and the three bright excitons with transition dipoles along the X, Y, Z directions, respectively:[9]

$$\langle \mathcal{O}|\, \psi\rangle = \tilde{M}^{-1} \langle P|\, \psi\rangle, \tag{S10}$$

where the unitary transformation matrix $\tilde{M} = \langle \mathcal{O}|P\rangle$ is,

$$\tilde{M} = \begin{pmatrix} 0 & -\frac{1}{\sqrt{2}} & \frac{i}{\sqrt{2}} & 0 \\ -\frac{1}{\sqrt{2}} & 0 & 0 & \frac{1}{\sqrt{2}} \\ \frac{1}{\sqrt{2}} & 0 & 0 & \frac{1}{\sqrt{2}} \\ 0 & \frac{1}{\sqrt{2}} & \frac{i}{\sqrt{2}} & 0 \end{pmatrix} . \tag{S11}$$

In this basis, the ground exciton wavefunction of a NPL from Eq. (3) for each X_i state can be rewritten to

$$\Psi_{d,X_i}(\boldsymbol{r}_e, \boldsymbol{r}_h) = \frac{4\,|X_i\rangle}{d\sqrt{L_x L_y}} \cos\left(\frac{\pi X}{L_x}\right) \cos\left(\frac{\pi Y}{L_y}\right) \cos\left(\frac{\pi z_e}{d}\right) \cos\left(\frac{\pi z_h}{d}\right) \phi_d(\boldsymbol{r}_e - \boldsymbol{r}_h) \ . \tag{S12}$$

Averaging the SR exchange operator described in Eq. (9) over the total wavefunction from Eq. (S12) we arrive at Eqs. (12) and (13).

4. Long-range exchange

To find the magnitude of the LR exchange interaction using Cho's approach described in Eqs. (14) and (15), we need to find the unit cell momentum matrix element, which can be written as

$$\boldsymbol{p}_{X_i} = P_{cv}\tilde{\boldsymbol{p}}_{X_i} \ , \tag{S13}$$

where $\tilde{\boldsymbol{p}}_{X_i}$ is a dimensionless vectorial quantity and P_{cv} is the Kane matrix element $P_{cv} = -i\langle s|P|Z\rangle$. We used the relation between matrix elements of the electric dipole and the momentum operators, $\langle 1|\hat{\boldsymbol{\mu}}|2\rangle = i(e\hbar/m_0)\langle 1|\hat{\boldsymbol{p}}|2\rangle/(E_2-E_1)$, and have defined the transition energy as $E_2 - E_1 = \hbar\omega$. Note that $|\tilde{\boldsymbol{p}}_{X_i}| = \sqrt{2/3}$ for each of the bright exciton states in a cubic perovskite.

With expressions for the exciton envelope wavefunctions $f(\boldsymbol{r}_e, \boldsymbol{r}_h)$, we can thus calculate the polarization and then evaluate the integral in Eq. (14). For excitons states represented in the D, X, Y, Z basis the transition dipoles are aligned to the crystal axes.

$$\boldsymbol{\mathcal{P}}_{X_i}(\boldsymbol{r}) = if(\boldsymbol{r}_e, \boldsymbol{r}_e)|\tilde{\boldsymbol{p}}_{X_i}|\left(\frac{P_{cv}\hbar e}{m_0\hbar\omega}\right)\hat{\boldsymbol{n}}_{X_i} \ , \tag{S14}$$

where the unit vector $\hat{\boldsymbol{n}}_{X_i}$ is the unit vector giving the direction of the dipole for exciton X_i. Then Eq. (14) for the LR exchange correction can be recast as

$$H^{LR}_{X_i} = |\tilde{\boldsymbol{p}}_{X_i}|^2\frac{1}{\epsilon_\infty}\left(\frac{P_{cv}\hbar e}{m_0\hbar\omega}\right)^2\mathcal{I}_{X_i} \ , \tag{S15}$$

where the the LR exchange integral $\mathcal{I}_{X_i}$ is given by

$$\mathcal{I}_{X_i} = \int_{V_1} dV_1 \int_{V_2} dV_2 \left[-\boldsymbol{\nabla}_1 \cdot f(\boldsymbol{r}_1, \boldsymbol{r}_1)\hat{\boldsymbol{n}}_{X_i}\right]^* \frac{1}{|\boldsymbol{r}_1 - \boldsymbol{r}_2|}\left[-\boldsymbol{\nabla}_2 \cdot f(\boldsymbol{r}_2, \boldsymbol{r}_2)\hat{\boldsymbol{n}}_{X_i}\right] \ . \tag{S16}$$

The expression Eq. S15 can be rewritten in terms of the LT splitting, $\hbar\omega_{LT}(d)$,[10–12] the exchange overlap factor, Θ, from Eq. (11), and the dimensionless anisotropy function $\mathcal{A}_{X_i}$ defined as

$$\mathcal{A}_{X_i} = \frac{3}{4\pi}\frac{\Omega}{\Theta}\mathcal{I}_{X_i} \ . \tag{S17}$$

This leads to Eq. (16):

$$\Delta E_{X_i,LR} = \frac{\hbar\omega_{LT}(d)}{2}\tilde{f}_{X_i}\mathcal{A}_{X_i}\left(\frac{\Theta}{\Theta_{\text{bulk}}}\right) \ , \tag{S18}$$

where $\tilde{f}_{X_i} = |\tilde{\boldsymbol{p}}_{X_i}|^2$ and the bulk exchange overlap $\Theta_{\text{bulk}} = \Omega/(\pi a_x^3)$ is given in terms of unit cell volume Ω and the bulk exciton radius a_x. The dimensionless parameters $\mathcal{A}_{X_i}$ correspondingly depend on the ratio of NPL thickness to the lateral size, d/L, since the LR exchange involves out-of-plane derivatives.

To find the dimensionless functions $\mathcal{A}_X(d/L)$ and $\mathcal{A}_Z(d/L)$ for the ground exciton state in the NPL, we can rewrite the envelope function of the exciton ground state. Considering COM coordinates X and Y, electron coordinates $\boldsymbol{r}_e$, and hole coordinates $\boldsymbol{r}_h$, the envelope function becomes:

$$\Psi^d_{\text{NPL}}(\boldsymbol{r}_e,\boldsymbol{r}_h) = \frac{4}{d\sqrt{L_xL_y}}\cos\left(\frac{\pi X}{L_x}\right)\cos\left(\frac{\pi Y}{L_y}\right)\cos\left(\frac{\pi z_e}{d}\right)\cos\left(\frac{\pi z_h}{d}\right)\phi_d(\boldsymbol{r}_e-\boldsymbol{r}_h) \ . \tag{S19}$$

Consequently, the polarization becomes

$$\begin{aligned}\boldsymbol{\mathcal{P}}_{X_i}(\boldsymbol{r}) &= i\Psi^d_{\text{NPL}}(\boldsymbol{r}_e,\boldsymbol{r}_h)|\tilde{\boldsymbol{p}}_{X_i}|\left(\frac{P_{cv}\hbar e}{m_0\hbar\omega}\right)\hat{\boldsymbol{n}}_{X_i} \\ &= i\left(\frac{P_{cv}\hbar e}{m_0\hbar\omega}\right))|\tilde{\boldsymbol{p}}_{X_i}|\frac{4}{d\sqrt{L_xL_y}}\cos\left(\frac{\pi X}{L_x}\right)\cos\left(\frac{\pi Y}{L_y}\right)\cos^2\left(\frac{\pi z}{d}\right)\phi_d(0)\hat{\boldsymbol{n}}_{X_i} \ .\end{aligned} \tag{S20}$$

Using this expression in Eq. (S15) we can now calculate the LR exchange corrections. The induced charge density for a square NPL ($L_x = L_y = L$) is proportional to

$$f(X,Y,z) = \frac{4}{Ld}\phi_d(0)\cos\left(\frac{\pi X}{L}\right)\cos\left(\frac{\pi Y}{L}\right)\cos^2\left(\frac{\pi z}{d}\right) \ , \tag{S21}$$

and allows us to rewrite the LR exchange splitting:

$$\Delta E_{X_i,LR} = \tilde{f}_{X_i}\frac{\hbar\omega_{LT}(d)}{2}\frac{3}{4\pi}\pi a_x^3\int_{V_1}dV_1\int_{V_2}dV_2\frac{[df(X_1,Y_1,z_1)/dX_{i,1}]^*\,[df(X_2,Y_2,z_2)/dX_{i,2}]}{\sqrt{(X_1-X_2)^2+(Y_1-Y_2)^2+(z_1-z_2)^2}} \ . \tag{S22}$$

Introducing dimensionless coordinates $X' = X/L$, $Y' = Y/L$, and $z = z/d$ we can express the LR exchange splitting via dimensionless functions $\mathcal{A}_X(r)$ and $\mathcal{A}_z(r)$ where $r = d/L$:

$$\Delta E_{Z,LR} = \tilde{f}_Z\frac{\hbar\omega_{LT}(d)}{2}\frac{3}{2d}\pi a_x^3\left|\phi_d(0)\right|^2\mathcal{A}_Z(r) \ , \tag{S23}$$

$$\Delta E_{X,LR} = \Delta E_{Y,LR} = \tilde{f}_X\frac{\hbar\omega_{LT}(d)}{2}\frac{3}{2d}\pi a_x^3\left|\phi_d(0)\right|^2\mathcal{A}_X(r) \ . \tag{S24}$$

The functions $\mathcal{A}_X(r)$ and $\mathcal{A}_Z(r)$ are given by the integrals

$$\mathcal{A}_Z = \frac{8\pi}{r} \int_{-1/2}^{1/2} dX_1' dY_1' dz_1' dX_2' dY_2' dz_2' \; \frac{\cos(\pi X_1')\cos(\pi Y_1')\sin(2\pi z_1')\cos(\pi X_2')\cos(\pi Y_2')\sin(2\pi z_2')}{\sqrt{(X_1'-X_2')^2+(Y_1'-Y_2')^2+r^2(z_1'-z_2')^2}} \,,$$

$$\mathcal{A}_X = 8\pi r \int_{-1/2}^{1/2} dX_1' dY_1' dz_1' dX_2' dY_2' dz_2' \; \frac{\sin(\pi X_1')\cos(\pi Y_1')\cos^2(\pi z_1')\sin(\pi X_2')\cos(\pi Y_2')\cos^2(\pi z_2')}{\sqrt{(X_1'-X_2')^2+(Y_1'-Y_2')^2+r^2(z_1'-z_2')^2}} \,. \tag{S25}$$

These terms introduce an additional anisotropy of the LR exchange interaction to the one created by the Bloch functions and described by the factors $\tilde{f}_{Z,X}$. The multi-coordinate integration in Eq. (S25) was performed using the VEGAS+ algorithm for adaptive Monte Carlo integration,[13, 14] and the results are shown in Fig. 2b and Supporting Fig. 9.

5. Three-level decay model

Consider a set of exciton levels as illustrated in Figure 4(a): two bright levels B_1 and B_2 with degeneracy n_1 and n_2, higher in energy than a dark level D by ΔE_1 and ΔE_2 respectively, with $\Delta E_2 > \Delta E_1$. Let the zero-temperature relaxation rate from B_1 to D be γ_{1D}, from B_2 to D be γ_{2D}, and from B_2 to B_1 be γ_{21}. Let the rate of recombination from D to G be Γ_D, from B_1 to G be Γ_{B1} and from B_2 to G be Γ_{B2}. Transitions between two excited states with energy difference ΔE arise through emission or absorption of a phonon with energy ΔE. These phonons have population $N_B(\Delta E) = \left(\exp\left(\frac{\Delta E}{k_B T}\right) - 1\right)^{-1}$, so the transition rate is $\gamma_i N_B(\Delta E)$, where γ_i is the zero-temperature relaxation rate from the higher-energy to the lower-energy state. Let $N_{B1} = N_B(\Delta E_1)$, $N_{B2} = N_B(\Delta E_2)$, and $N_{B21} = N_B(\Delta E_2 - \Delta E_1)$. Transitions into a state with degeneracy n are enhanced by a factor of n.

Let the populations of the levels be p_{B1}, p_{B2}, p_D. After initial excitation, the population of D evolves according to:

$$\frac{dp_D}{dt} = -(\underbrace{\Gamma_D}_{D\downarrow G} + \underbrace{n_1\gamma_{1D}N_{B1}}_{D\uparrow B_1} + \underbrace{n_2\gamma_{2D}N_{B2}}_{D\uparrow B_2})p_D + \underbrace{\gamma_{1D}(N_{B1}+1)p_{B1}}_{B_1\downarrow D} + \underbrace{\gamma_{2D}(N_{B2}+1)p_{B2}}_{B_2\downarrow D} \,. \tag{S26}$$

Similarly

$$\frac{dp_{B1}}{dt} = \underbrace{n_1\gamma_{1D}N_{B1}p_D}_{D\uparrow B_1} - [\underbrace{\Gamma_{B1}}_{B_1\downarrow G} + \underbrace{\gamma_{1D}(N_{B1}+1)}_{B_1\downarrow D} + \underbrace{n_2\gamma_{21}N_{B21}}_{B_1\uparrow B_2}]p_{B1} + \underbrace{n_1\gamma_{21}(N_{B21}+1)p_{B2}}_{B_2\downarrow B_1} \ , \tag{S27}$$

$$\frac{dp_{B2}}{dt} = \underbrace{n_2\gamma_{2D}N_{B2}p_D}_{D\uparrow B_2} + \underbrace{n_2\gamma_{21}N_{B21}p_{B1}}_{B_1\uparrow B_2} - [\underbrace{\Gamma_{B2}}_{B_2\downarrow G} + \underbrace{\gamma_{2D}(N_{B2}+1)}_{B_2\downarrow D} + \underbrace{n_1\gamma_{21}(N_{B21}+1)}_{B_2\downarrow B_1}]p_{B2} \ . \tag{S28}$$

Let

$$\mathbf{p} = \begin{pmatrix} p_D \\ p_{B1} \\ p_{B2} \end{pmatrix} \ . \tag{S29}$$

The system of differential equations for the populations may be written as

$$\frac{d\mathbf{p}}{dt} = \mathcal{M}\mathbf{p} \ , \tag{S30}$$

where the matrix $\mathcal{M}$ is

$$\mathcal{M} = \begin{pmatrix} -\Gamma_D - n_1\gamma_{1D}N_{B1} - n_2\gamma_{2D}N_{B2} & \gamma_{1D}(N_{B1}+1) & \gamma_{2D}(N_{B2}+1) \\ n_1\gamma_{1D}N_{B1} & -\Gamma_{B1} - \gamma_{1D}(N_{B1}+1) - n_2\gamma_{21}N_{B21} & n_1\gamma_{21}(N_{B21}+1) \\ n_2\gamma_{2D}N_{B2} & n_2\gamma_{21}N_{B21} & -\Gamma_{B2} - \gamma_{2D}(N_{B2}+1) - n_1\gamma_{21}(N_{B21}+1) \end{pmatrix} . \tag{S31}$$

This general solution to this linear ODE is

$$\mathbf{p}(t) = \mathbf{v}_1 e^{\lambda_1 t} + \mathbf{v}_2 e^{\lambda_2 t} + \mathbf{v}_3 e^{\lambda_3 t} \ , \tag{S32}$$

where λ_i are the eigenvalues of $\mathcal{M}$ and $\mathbf{v}_i$ are the corresponding eigenvectors. We are interested in the decay rates λ_i. The expressions for λ_i are too complicated to write down fruitfully, but they can still be used for fitting.

We now proceed to fit the model to experiment, identifying the λ_i with the smallest magnitude as the long decay rate. The full model has 8 parameters, too many to produce a reliable fit. In order to reduce the number of parameters, we fix ΔE_1 and ΔE_2 to the calculated values. The ratio of the radiative decay rates Γ_{B1}/Γ_{B2} can also be calculated. The rate is proportional to the square of the transition matrix element, which in turn is proportional to f_{X_i} .[10, 11] In the presence of dielectric confinement, the Z rate is further reduced because the electric field of the photon is screened by the dielectric constant of the medium ε_o= 2.13 rather than the internal $\varepsilon_i{}^{\infty} = 4.76$. Since the interaction strength is

proportional to the square of the electric field, Γ_Z includes an additional factor of $(\varepsilon_o/\varepsilon_i{}^\infty)^2$. Hence, we make the approximation

$$\frac{\Gamma_Z}{\Gamma_{XY}} = \frac{f_z}{f_{xy}} \left(\frac{\varepsilon_o}{\varepsilon_i{}^\infty}\right)^2 . \tag{S33}$$

Fixing ΔE_1, ΔE_2, and Γ_{B1}/Γ_{B2} reduces the fit to a much more reasonable 5 parameters. Fig. 3(a) shows that the calculated ΔE values are in good agreement with the spectral shift data, and the good agreement between the fitted decay curves and the experimental data in Fig. 4(c) shows that the calculated values are consistent with the decay measurements as well.

This model also allows the prediction of steady-state photoluminescence. Since the pump energy is high above the exciton levels, we assume the pump populates the levels equally. Therefore the steady-state population $\mathbf{p}^{ss}$ is given by

$$\mathbf{p}^{\text{pump}} + \mathcal{M} \cdot \mathbf{p}^{ss} = 0, \qquad \text{where} \qquad \mathbf{p}^{\text{pump}} = \begin{pmatrix} 1/4 \\ 1/4 \\ 1/4 \end{pmatrix} . \tag{S34}$$

The photoluminescence at energy E is given by a sum over states:

$$PL(E) = \sum_i n_i p_i^{ss} \Gamma_i N_{E_i,\sigma_i}(E) \tag{S35}$$

where the lineshape of state i is described by a normal distribution $N_{E_i,\sigma_i}(E)$ with standard deviation σ_i centered at the energy of the state E_i. For comparison with the experimental temperature-resolved photoluminescence (Supporting Fig. 3), theoretical temperature-resolved photoluminescence maps are also generated (Supporting Fig. 13). The PL is normalized to integrate to 1 at each temperature, as is done in the experiments. The only adjustable parameters in Supporting Fig. 13 are the standard deviations σ_i.

6. Hybrid DFT calculations of nanoplatelet electronic structure

Density functional theory calculations are based on the HSE functional [15] using projector-augmented wave (PAW) psuedopotentials,[16] as implemented in the VASP code.[17] We use 35% Hartree-Fock mixing and a screening parameter of 0.1 Å^{-1} in order to correct for the band-gap error inherent to standard implementations of DFT.[10]

Spin-orbit coupling is included for all calculations. Our nanoplatelets use the cubic phase of $CsPbBr_3$, 1x1 in the lateral directions. Out of plane, the (100) surfaces are terminated in the $PbBr_2$ plane, with at least 15 Å of vacuum to avoid surface-surface interactions. Atomic relaxations of the outermost layers are included. We use a 400 eV plane-wave cutoff, together with a 6x6x1 k-point mesh.

The calculated band gaps are shown in Supporting Fig. 14. The calculated bulk band gap of cubic $CsPbBr_3$ is 1.75 eV (indicated with the dashed orange line in Supporting Fig. 14). As expected, due to quantum confinement the band gaps of the nanoplatelets are larger, and increase with decreasing thickness. The calculated trend is in good agreement with the experimental observations (in blue), but the absolute value of the band gap is underestimated, possibly due to the choice of crystal structure or the lack of surface ligands in the calculations. This may largely be attributed to the difference between the cubic and tetragonal band gaps for the 3D system.[10] We therefore shift the calculated gaps upwards by 0.6 eV, equal to the difference between the calculated bulk band gap and the value used in the effective mass modeling ($E_g = 2.342$ eV), obtaining the data shown in yellow dotted and dashed lines. After this shift, the agreement with the experimental trend is clear.

We have also determined the effective masses of electrons and holes in the nanoplatelets, which do not change considerably in comparison with the bulk $CsPbBr_3$ band structure. From HSE+SOC calculations of bulk $CsPbBr_3$, both m_e and m_h are 0.16. In the 2 ML nanoplatelet (the thinnest calculated here), m_h is 0.17, while m_e is 0.23. The higher increase of m_e may be due to the undercoordinated surface Pb atoms, which will affect the nanoplatelet m_e since Pb p orbitals dominate the character of the conduction-band minimum. Nevertheless, the similarity between bulk and nanoplatelet effective masses provides reassurance that the effective-mass approximation remains valid even for the thinnest $CsPbBr_3$ nanoplatelets.

	$PbBr_2$ (ml)	Cs-oleate (μl)	Acetone (ml)
2 ML	3	150	2
3 ML	1.5	150	2
4 ML	1.3	150	2
5 ML	1.2	150	2
6 ML	1	150	2
8 ML	0.8	150	2

Supporting Table 4. Precursor and acetone amounts for different thicknesses.

SUPPORTING REFERENCES

[1] D. Rossi, X. Liu, Y. Lee, M. Khurana, J. Puthenpurayil, K. Kim, A. V. Akimov, J. Cheon, D. H. Son, *Nano Lett.* **2020**, *20* 7321.

[2] M. Gramlich, C. Lampe, J. Drewniok, A. S. Urban, *J. Phys. Chem. Lett.* **2021**, *12* 11371.

[3] N. C. Ritova, *Vestnik of Moskow University* **1967**, *N3* 30, arXiv:1806.00976 [cond-mat.mes-hall].

[4] L. V. Keldish, *JETP Lett.* **1979**, *29* 716.

[5] E. Hanamura, N. Nagaosa, *Mater. Sci. Eng. B* **1988**, *1* 255.

[6] X. Hong, T. Ishihara, A. U. Nurmikko, *Phys. Rev. B.* **1992**, *45* 6961.

[7] Z. Yang, A. Surrente, K. Galkowski, A. Miyata, O. Portugall, R. J. Sutton, A. A. Haghighirad, H. J. Snaith, D. K. Maude, P. Plochocka, R. J. Nicholas, *ACS Energy Lett* **2017**, *2* 1621.

[8] A. L. Efros, M. Rosen, *Annu. Rev. Mater. Sci* **2000**, *30* 475.

[9] P. C. Sercel, Z. V. Vardeny, A. L. Efros, *Nanoscale* **2020**, *12* 18067.

[10] P. C. Sercel, J. L. Lyons, D. Wickramaratne, R. Vaxenburg, N. Bernstein, A. L. Efros, *Nano Lett.* **2019**, *19* 4068.

[11] P. C. Sercel, J. L. Lyons, N. Bernstein, A. L. Efros, *J. Chem. Phys* **2019**, *151* 234106.

[12] B. D. Folie, J. A. Tan, J. Huang, P. C. Sercel, M. Delor, M. Lai, J. L. Lyons, N. Bernstein, A. L. Efros, P. Yang, N. S. Ginsberg, *J. Phys. Chem. A* **2020**, *124* 1867.

[13] G. P. Lepage, *J. Comput. Phys.* **1978**, *27* 192.

[14] G. P. Lepage, *J. Comput. Phys.* **2021**, *439*.

[15] J. Heyd, G. E. Scuseria, M. Ernzerhof, *J. Chem. Phys.* **2003**, *118* 8207.

[16] P. E. Blöchl, *Phys. Rev. B* **1994**, *50* 17953.

[17] G. Kresse, J. Fürthmuller, *Phys. Rev. B* **1996**, *54* 11169.
